# Evaluating Pt Nanoparticle Electrocatalyst Stability on Corrosion Free Carbon Supports Using Identical Location TEM

**DOI:** 10.1002/smll.202513549

**Published:** 2026-07-24

**Authors:** Daniel Houghton, Yisong Han, Richard Beanland, Louis Godeffroy, Frédéric Kanoufi, Viacheslav Shkirskiy, Jonathan Sharman, Julie V. Macpherson

**Affiliations:** ^1^ Department of Chemistry University of Warwick Coventry UK; ^2^ Department of Physics University of Warwick Coventry UK; ^3^ CNRS ITODYS Université Paris Cité Paris France; ^4^ Johnson Matthey Technology Centre Reading UK

**Keywords:** accelerated stress test, boron doped diamond, identical location‐transmission electron microscopy, platinum nanoparticles, TEM image analysis

## Abstract

Probing platinum (Pt) nanoparticle (NP) stability, free from convolution with corrosion of the underlying carbon support, under the start‐stop conditions of a proton exchange membrane fuel cell, is challenging. To address this problem with a focus on tracking NP morphology changes, we use identical‐location transmission electron microscopy, in combination with corrosion‐resistant, electron‐transparent, boron‐doped diamond electrodes. Automated image analysis is developed to enable faster and easier processing. Information concerning NP area, positional changes, and relationship to nearest neighbor distance is extracted on an NP‐by‐NP basis by tracking the same NPs, pre‐ and post‐accelerated stress testing in perchloric acid under conditions which promote carbon corrosion for sp^2^ carbons. Measurements are made in several locations with ca. 200 NPs analyzed per image. A significant fraction of the NPs, 40%–50%, are found to be area stable, with only a small number growing and the remaining decreasing very slightly in area (less than a single atom layer) due to transient dissolution. The average distance of travel of an NP after accelerated stress testing is ∼0.36 nm (*n* = 545). This data highlights both the stability of the Pt‐BDD interaction and the potential for BDD use as an electrocatalyst support, under high anodic potentials.

## Graphical abstract

Pt nanoparticle stability during accelerated stress testing at high anodic potentials was investigated using identical location TEM on a corrosion‐resistant boron‐doped diamond (BDD) electrode. Changes in nanoparticle size, position, and response with respect to nearest neighbor distance on a particle‐by‐particle basis were tracked using machine vision analysis. The images indicated high stability of the nanoparticles on the BDD.

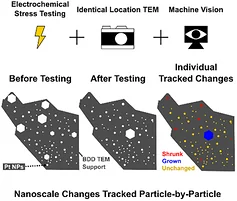

## 1 Introduction

Platinum (Pt) is an important metal for electrocatalysis, especially for energy production, where it is commonly employed as an electrocatalyst in proton exchange membrane fuel cells (PEMFCs) due to its enhanced stability in highly acidic media [1]. In the PEMFC, Pt is used to catalyze both the anode hydrogen oxidation reaction and the cathode oxygen reduction reaction (ORR) [2]. Pt is especially important for ORR as it facilitates the 4‐electron reduction of oxygen to water [2, 3, 4]. In the PEMFC, Pt is used in nanoparticle (NP) form and is supported on high surface area, aggregated carbon black (CB) particles [1, 5]. CB, which is a form of graphitized carbon [6], has many advantages as an electrocatalyst support; it is cheap to manufacture on large scales and has an extremely high surface area (e.g., 800 m^2^g^−1^) [7]. However, a significant issue with Pt NP/CB electrodes is their limited operational lifetime due to a variety of degradation mechanisms associated with the Pt NPs or the carbon support. For the Pt NPs, these include aggregation (coalescence and migration), Ostwald ripening, and loss of NPs from the surface via detachment or dissolution. For the carbon support, corrosion is the main issue [5, 8, 9].

Carbon corrosion occurs via a kinetically slow electro‐oxidative pathway (Equation 1), likely due to passivating surface oxide formation [10].

(1) $$C\;+2H_{2}O\to CO_{2}+4H^{+}+4e^{-}\to E^{0}=0.207V\;\mathrm{vs}.\;\mathrm{RHE}$$

However, if the potential rises above approximately 1 V vs. RHE, the rate of carbon corrosion increases [5], which is further exacerbated by temperature and the presence of Pt [5]. Such potentials are experienced during the start‐up and shut‐down procedures of a PEMFC [5]. To simulate real‐life fuel cells, accelerated stress tests (ASTs) are used to mimic different fuel cell operational conditions on a much shorter timescale (hours instead of years). They consist of thousands of electrochemical cycles over appropriate potential ranges [5]. To examine the behavior of the cell under carbon corrosion AST conditions, which also replicates the start/stop process of a fuel cell, the potential is typically cycled between 1.0 and 1.5 V vs. RHE [11]. Previous studies to assess Pt stability at potentials >1.0 V vs. RHE have used polycrystalline Pt disks and foils [8, 12], as well as supported Pt NPs [13]. Typical measurements range from the macroscale e.g., monitoring the dissolution of Pt using inductively‐coupled plasma mass spectrometry (ICP‐MS) [8], assessing changes in the electrochemical active surface area [14], to nanoscale microscopy measurements.

Moving to corrosion‐resistant carbon supports is not only useful for PEMFCs but also for water electrolyzers [15]. For the latter, traditional carbon supports cannot be employed with oxygen evolution reaction (OER) electrocatalysts due to the higher potentials associated with OER [16]. Polycrystalline boron‐doped diamond (BDD), which is metallically doped (i.e., >10^20^ B atoms per cm^3^) [17], has been shown to be corrosion resistant under conditions pertinent to both start/stop PEMFC operation and OER [18, 19]. Although as a note of caution, it is important to assess the material quality first as BDD growth conditions can result in sp^2^ carbon formation [17, 20] which increases susceptibility towards anodic corrosion [21].

Transmission electron microscopy (TEM) is a useful tool in the study of metal NP electrocatalysts [22]. *Operando* electrochemical (EC)‐TEM enables TEM measurements to be made in real‐time in the measurement solution [23]. In contrast, ex situ EC‐TEM images the surface (under vacuum), post‐electrochemical treatment and after removal from the electrolyte, with the advantage of enabling higher‐resolution imaging, tomography and analytical electron microscopy free from solvent and supporting electrolyte effects [24, 25, 26, 27, 28]. Identical location (IL) measurements enable tracking of the same NPs before and after EC treatment and have been used to investigate NP electrocatalytic degradation mechanisms [24, 25, 26, 27, 28]. The focus is typically on Pt or Pt alloy NPs supported on thin‐film amorphous carbon on Au mesh TEM finder grids, either used as is or attached to CB [25, 26, 27, 28]. When higher electrochemical cycling potentials are employed, corrosion of the gold grid, gold redeposition [29], and carbon film damage [18] have all been reported [18, 29, 30].

Analysis of NP microscopy images before and after AST treatment has largely been by manual inspection of NP size and shape, for both IL [27, 28] and non‐IL imaging [31, 32, 33]. However, this makes image analysis time‐consuming and often difficult. As a result, TEM data sets, which are often information‐dense, can be under‐analyzed, leaving significant amounts of data wasted. To remedy this, there has been a recent move toward the use of more advanced image analysis techniques. For example, work into evaluating nanoscale changes in fuel cell NPs employed automated data acquisition and analysis methods to evaluate changes in NP size distributions over tens of thousands of particles [34]. However, whilst useful at providing statistically relevant data, it was not possible to correlate the response of individual particles, pre‐ and post‐treatment.

In electron microscopy, algorithms for image recognition and analysis (“computer/machine vision”) that enable the computer to “see” and compare images are becoming more commonplace [24, 33, 35, 36, 37, 38, 39]. These approaches convert the information within images into measurable descriptors, such as the distribution of particle size and shape, trajectories, and defect characteristics, such that structural evolution can be correlated with electrochemical history. For example, such methods have been used to track atom‐level structural changes in tens of Pt/Co NPs catalysts supported on CB subjected to potential cycling [24]. Further developments include automatic detection, segmentation, and classification of NPs via e.g., the AutoDetect‐mNP algorithm [40]. Complete pipeline workflows have also been proposed [41], which are used to extract restructuring pathways via automated multi‐object tracking, on‐the‐fly (in real time), or even on latent representations [42]. In parallel, deep learning‐assisted segmentation/tracking is increasingly being employed to recognize features of importance, e.g. defect evolution using DefectTrack [43]. Furthermore, image recognition approaches can also be coupled with machine learning approaches to group complex datasets in ways a human might not see. For example, at the ultimate resolution limit, such methods have been used to track single‐atom (catalyst) sites in transition metal complexes from TEM images [33, 44] or to estimate, on the fly, atomic column heights from HRTEM images of NPs [45].

In the work described herein, we use IL‐EC‐TEM, in combination with machine vision methods, to track the individual response of ∼two hundred Pt NPs (per image) under carbon corrosion AST potential cycling conditions. The employment of negligible sp^2^ carbon content BDD as a BDD‐TEM substrate [18, 46] enables Pt NP stability to be probed under conditions where the response is free from simultaneous carbon corrosion [5, 11]. Moreover, it also allows examination of the feasibility of using BDD as a metal NP electrocatalyst support under anodic oxidative conditions. Image analysis is used to track changes in the position (center of intensity) and area of each NP on a particle‐by‐particle basis and identify any correlations between NP diameter change and nearest neighbor distance. Multiple locations on the same IL‐TEM substrate are imaged to increase the number of NPs undergoing statistical analysis.

## 2 Results and Discussion

To assess Pt NP stability under AST conditions pertinent to PEMFC start‐up and shutdown, IL‐TEM using a BDD TEM substrate was used to directly compare the pixel area (size) of Pt NPs on a particle‐by‐particle basis before and after AST electrochemical cycling. The 3 mm diameter circular BDD TEM substrate was fabricated using ion milling to thin the disk from edge to center until a small (∼200 nm) hole in the center resulted (see Experimental Section). Surrounding the hole is a ∼35 µm region of electron beam transparent BDD [18]. The BDD is oxygen‐terminated and presents a dominant (110) crystallography [18, 47]. The Pt NPs on the BDD TEM substrate were prepared using sputter coating (Experimental Section), which ensures repeatable production of Pt NPs on the BDD‐TEM electrodes [48]. The electrochemical experimental set‐up is shown in Section S1.

**FIGURE 1:**
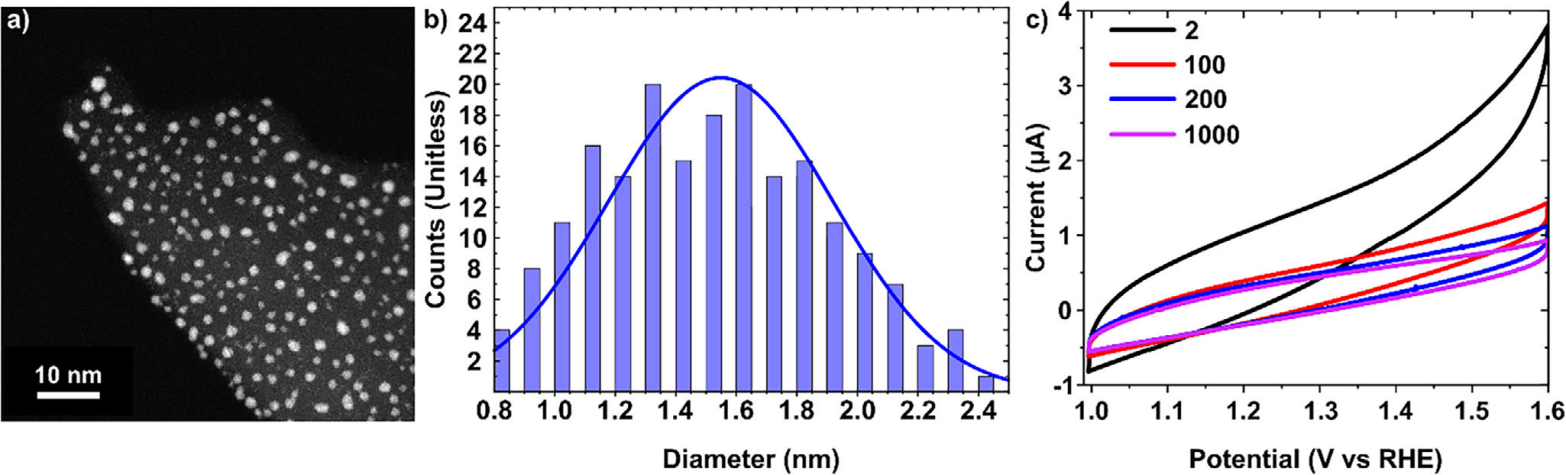
(a) ADF‐STEM image of the BDD TEM substrate coated with Pt NPs “Before AST”. (b) Particle size distribution histogram “Before AST”. (c) AST consisting of 1000 cycles from 1.0 to 1.6 V vs. RHE, in 0.5 M perchloric acid at a scan rate of 0.1 V s^−1^: cycles 2, 100, 200, and 1000 are shown.

Figure 1a is a high‐magnification annular dark‐field scanning TEM (ADF‐STEM) image of a section of the BDD TEM substrate in the electron beam transparent region surrounding the hole area [18]. The black region is the vacuum, whilst the remaining feature is the jagged hole coastline and associated electron beam transparent region of the Pt NP coated BDD. Figure 1b shows the NP size distribution histogram “Before AST”, using the image analysis approach described in the Experimental Section. Objects with diameters below 0.8 nm in diameter have been excluded in order to focus only on NPs for this study. 0.8 nm represents the diameter of the smallest NP, corresponding to a one shell polyhedron Pt NP, consisting of thirteen Pt atoms, twelve of which are surface atoms [49]. The data show a mean NP diameter of 1.6 ± 0.4 nm (*n* = 190 NPs, error measured by 1 standard deviation). Although NPs < 2 nm are present in PEMFCs [50], they exist in smaller numbers than those in the typical size range ∼2–5 nm diameter [51]. For the purpose of this study, however, they serve as a useful model system for the investigation of Pt NP stability on a BDD support, in a potential region where sp^2^ carbon corrosion would significantly contribute. Furthermore, with the NPs having a higher surface area to volume ratio, this could result in more significant changes being revealed.

Figure 1c shows the second, 100^th^, 200^th^, and 1000^th^ of 1000 CV cycles (1.0–1.6 V vs. RHE) for AST in 0.5 M perchloric acid using a freshly sputtered Pt NP BDD‐TEM electrode. For these studies, the Pt NPs were not electrochemically pre‐conditioned [52]. Previous TEM electron energy loss spectroscopy measurements of electrode thickness for the same material quality BDD under the same cycling conditions in acidic solution showed no evidence of corrosion (electrode thinning) [18]. Perchloric acid was specifically chosen in Figure 1c, as it is considered to be a non‐adsorbing/weakly adsorbing electrolyte [52]. This cycling region is expected to result in oxide formation (PtO_x_), but it is not sufficiently negative to desorb oxygen, reduce the Pt oxide or show H_ads/des_ features [8, 53]. The second cycle CV data is large but decreases to an almost constant state with increasing scan number. This is likely to be a convolution between PtOx formation and removal of surface‐bound hydrocarbon impurities  [52, 54], from sputtering or exposure to air [55]. Carbon contamination from sputtering can be observed even in high‐vacuum systems [56] and thus is likely when using a simple benchtop sputter coater to produce the Pt NPs.

**FIGURE 2:**
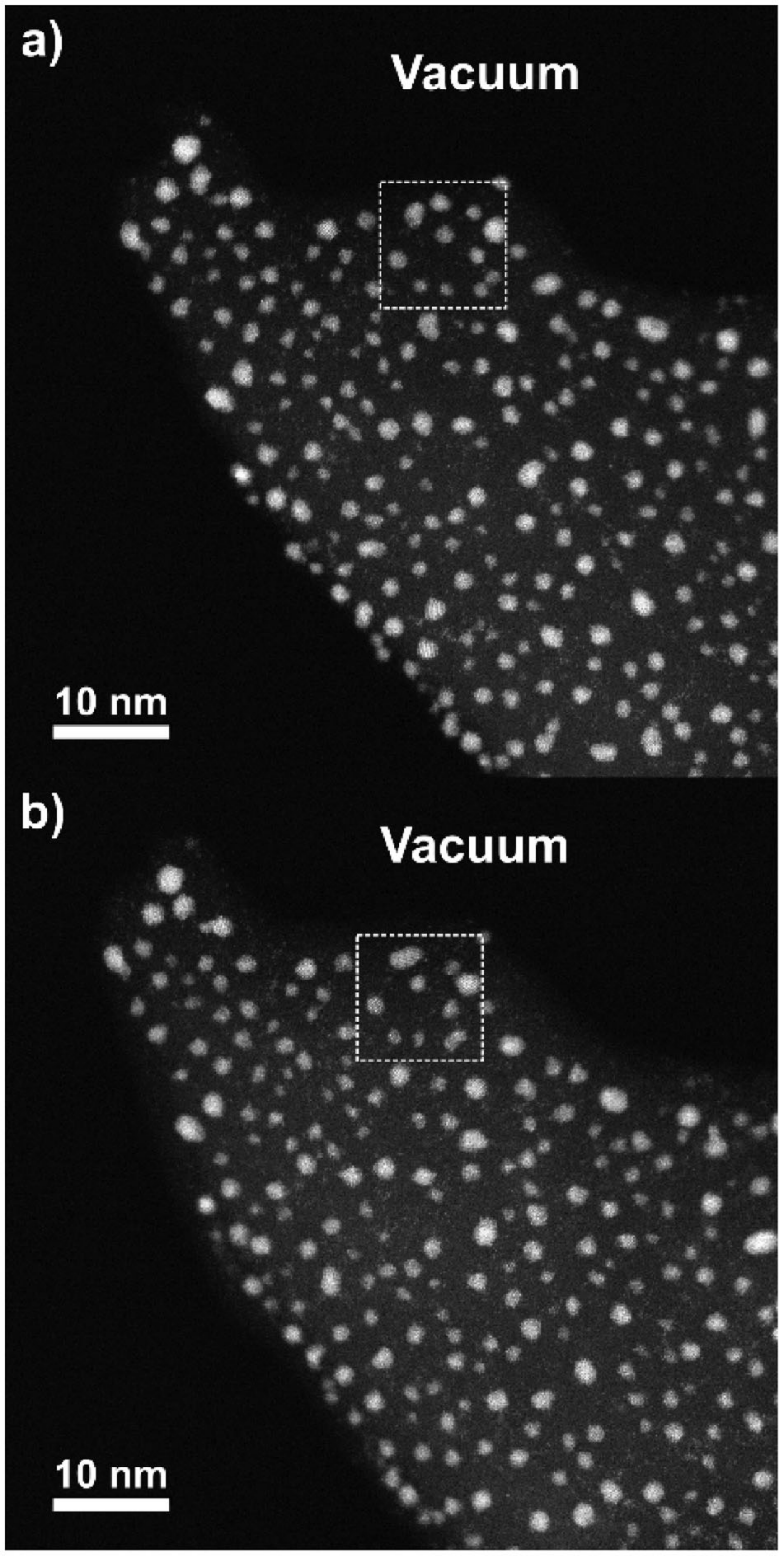
High‐magnification, identical‐location ADF‐STEM images of Pt NPs on a BDD TEM support before (a) and after (b) AST. An example area of interest is outlined with the dotted white square. The black background (vacuum) is the hole area of the BDD TEM substrate.

To evaluate size changes in the NPs as a result of AST, “Before AST” and “After AST” (1000 cycles) IL‐ADF‐STEM images were recorded as shown in Figure 2a,b, respectively. No recession in the BDD coastline, due to carbon corrosion [57], was observed when comparing “Before” and “After” images. In total, three sets of IL‐TEM before and after images were recorded in three different locations (see Section S2). Comparing Figure 2a,b, by eye, it is difficult to discern significant changes between the images, although some areas of Pt NP rearrangement are visually evident, for example, within the dotted box regions of Figure 2a,b. Electrochemical evaluation of the Pt NP‐BDD surface over a macroscopic electrode area, before and after AST, is discussed in Section S3. The data in Figure S3 support the presence of possible surface adsorbents prior to electrochemical AST, by revealing an increase in electrochemical Pt surface area post‐AST cycling. To explore changes to individual NPs on a particle‐by‐particle basis, in terms of changes in NPs' pixel area (size) and diameter, image analysis was implemented, as described in the Experimental Section. Diameters were extracted assuming the NPs are circular.

**FIGURE 3:**
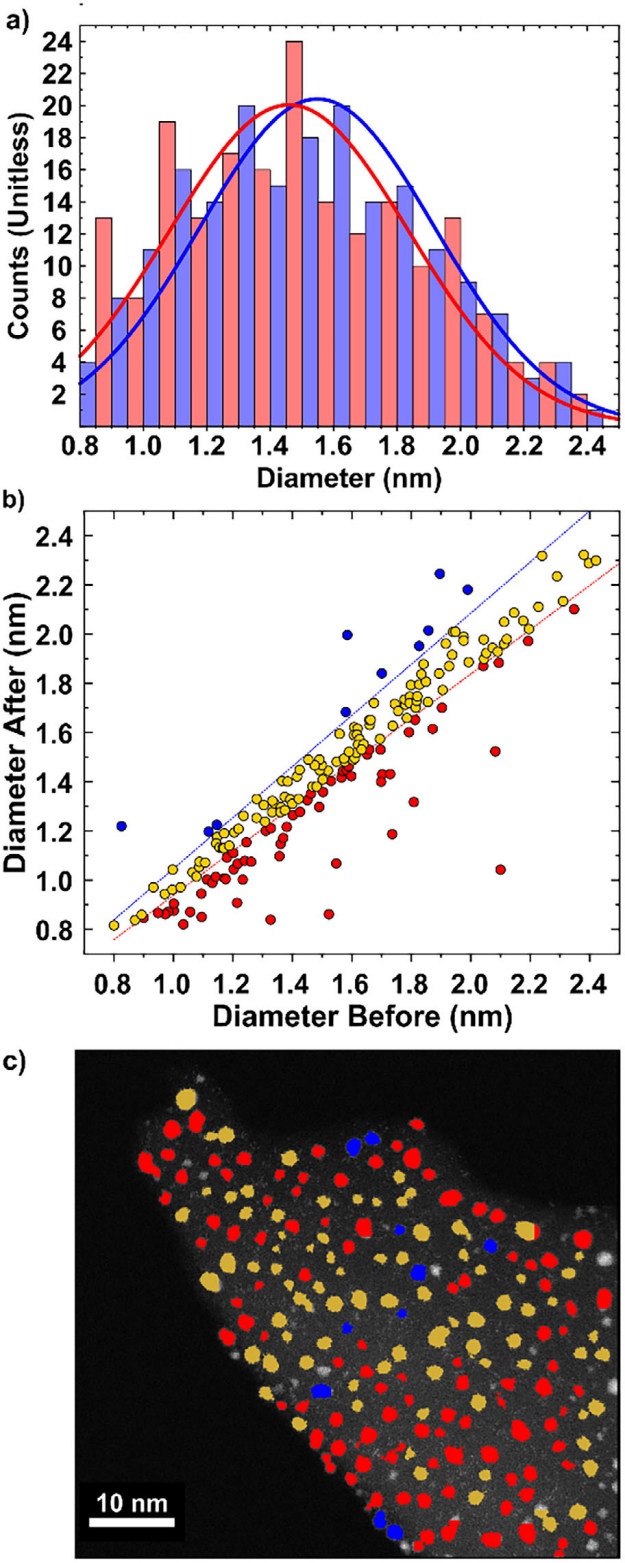
(a) Histogram of NP diameter with normal distribution plotted for “Before AST” (blue) and “After AST” (red). (b) Plot of “Before AST” Pt NP diameter vs. “After AST” NP diameter on a particle‐by‐particle basis. Red solid circles indicate NPs which have decreased in diameter, blue solid circles are NPs which have increased in diameter, and gold solid circles are NPs that are nominally “unchanged” within ± 5% measurement error (annotated with a +5% blue dash line and a −5% red dash line. (c) Annotated “Before AST” IL‐ADF‐STEM image where the individual NPs are colored in accordance with the color scheme in (b) to show those NPs that have increased (blue) /decreased (red) in NP diameter and those that have stayed the same (gold), within the measurement error, after AST.

A major benefit of IL‐TEM experiments is observing the size changes of individual NPs, not only the ensemble behavior. Figure 3a shows the NP diameter distribution histogram for the “After AST” image (red) alongside the “Before AST” image data (blue) for comparison. Figure 3a shows that, after AST cycling, the mean NP diameter has decreased from 1.6 ± 0.4 nm to 1.5 ± 0.4 nm (*n* = 190). Assuming a ± 5% measurement error, this difference is not significant. Further details on the calculation of measurement error are found in Section S4. Note that, as hydrocarbons have a very similar scattering profile to the BDD substrate, their effect on the measurement of Pt NP size, where Pt has a much higher atomic number and therefore a very strong ADF‐STEM signal, will be negligible. Furthermore, we do not see any evidence of significant contamination in the TEM images.

Figure 3b,c represent the data in a form where it is possible to see changes to the diameter of individual NPs post‐AST. Figure 3b shows a plot of NP diameter “Before AST” vs. NP diameter “After AST” for each NP. To help visualize changes in NP diameter that are not associated with possible errors introduced via the image analysis, ±5% error lines are introduced into Figure 3b. NPs that show an increase in diameter (outside this estimated error) are colored blue; 10 out of 189 NPs (5%) in Figure 3b. NPs that show no diameter change within the estimated error, 80 out of 189 NPs (42%), are colored gold, whilst those that show a diameter decrease (outside the estimated error) are colored red; 99 out of 189 NPs (52%). Figure 3c shows the “Before AST” IL‐TEM image color‐coded in the same way as Figure 3b. A small number of particles in Figure 3c are untracked; they retain the original TEM contrast color of gray. These are filtered out of the analysis method as a result of either (a) user‐imposed constraints, due to being < 0.8 nm in diameter and/or (b) the NP is too near the edge of the defined area of analysis (see Image Analysis of Experimental Section).

As Figure 3b shows, even in the case of a reduction in NP diameter, for almost all the NPs (∼90%), this is still only a minimal change of a ≤15% decrease in NP diameter (less than a single layer of Pt atoms). Figure S4 in Section S5 show the corresponding change in pixel area for each NP. The trends between Figure 3b and Figure S4 are very similar, giving confidence in the treatment of the NPs as circular in order to extract diameters.

During high‐magnification ADF‐STEM, depending on imaging conditions, the electron beam can induce NP movement, aggregation and growth [58, 59, 60]. Whilst imaging conditions were selected to minimize such effects [60], verification was important. Figure S6 in Section S6 contains two frames captured sequentially at the same magnification and imaging conditions used during IL‐ADF‐STEM experiments, but in the absence of electrochemical cycling. A total of 58 isolated NPs were selected, and the *x,y* position of their center of intensity was determined for both frames as described in Image Analysis (Experimental Section). The distance of movement of each NP from frame 1 to 2 is shown in Figures S6c,d via a histogram plot showing number of NPs vs. trajectory length. The mean and modal movement distances are 0.3 and 0.1 nm, respectively (a Pt atom has an atomic diameter of 0.27 nm), highlighting that electron beam‐induced movement effects on the sample are minimal.

**FIGURE 4:**
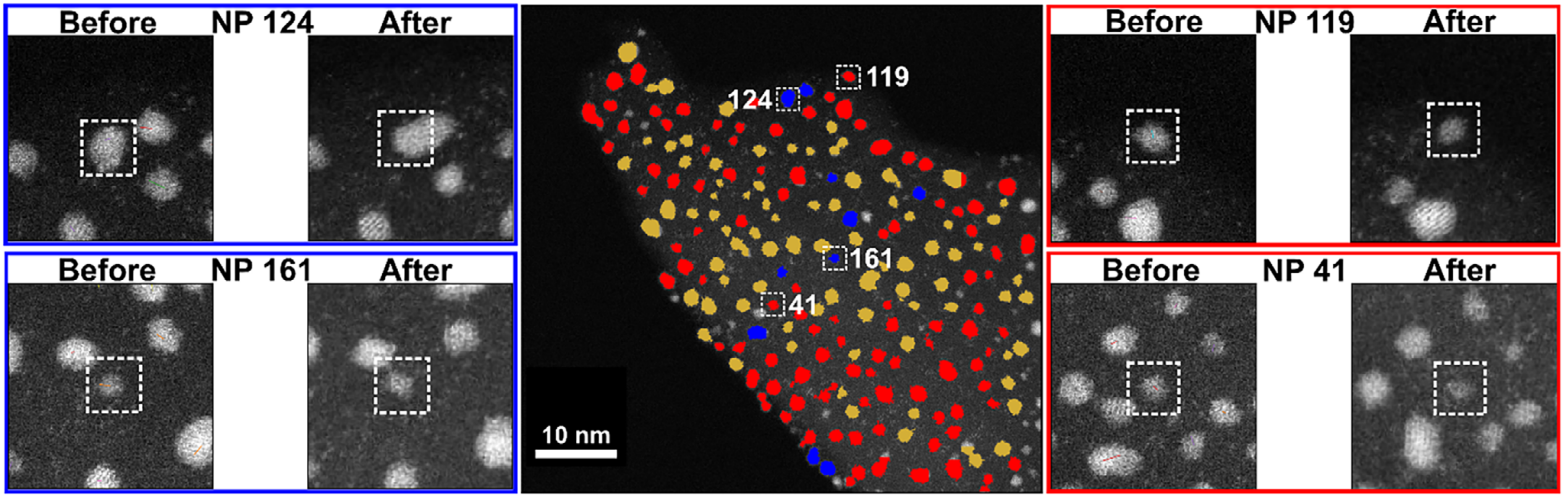
Annotated “Before AST” IL‐ADF‐STEM image where the individual NPs are colored in accordance with the color scheme in Figure 3. Two examples of shrinking particles (red, NPs 41 and 119) and two examples of growing particles (blue, NPs 124 and 161) are presented in the “Before” and “After” AST images. Image size for the four NPs is 9.6 nm × 9.6 nm.

Figure 4 and Figure S7, in Section S7, show higher‐magnification “Before” and “After” AST IL‐ADF‐STEM images of Pt NPs, which show a diameter increase. Based on visual inspection, four out of ten Pt NPs undergo coalescence with a NP neighbor (numbers 8, 123, 124, and 142). While more sophisticated AI‐based algorithms could be implemented in the future to detect such behaviors automatically [61], our goal here was not to remove the human eye from the analysis chain entirely, but rather to provide a simple and deterministic way to reduce the number of data points that needed to be inspected visually. The resulting data set could then serve as ground truth for future AI‐based algorithms. Coalescence of Pt NPs on CB under carbon corrosion AST conditions has been observed previously [5]. As corrosion and electron beam‐induced movement (Section S6) have been ruled out as possible causes here, our observation suggests that potential‐induced rearrangements and particle migration are possible under these anodic potential conditions. However, further experimental and computational work to validate this hypothesis is required, especially concerning the potential‐dependent interaction of Pt with the BDD surface.

Observation at higher resolution enables events not captured by the current method of image analysis to be revealed. For example, concerning the observed growth of NPs 111 and 173 (Figure S7), the nearest neighbor(s) appear to be amorphous clusters of atoms [60]. These are too small to constitute a NP and are therefore not analyzed in the “Before AST” image. However, they clearly play a part by disassembling and being consumed by the larger NP, evident in the “After AST” images. Such behavior resembles 2D and 3D Ostwald ripening, rather than coalescence, where nearby smaller clusters/particles feed the growth of larger NPs [14, 62]. NP 161 (Figure 4) is also interesting, as the “After AST” image shows an atom bridge forming between it and its nearest NP neighbor. The image analysis model has classified this particle as growing significantly. Further refinements to our image analysis models, e.g., atom counting [63], etc would be needed to help better identify such events. Atom bridges have been seen previously during gold NP electrochemical nucleation and growth [60], in situ heating of gold NPs [59], and electron beam induced NP growth [59].

Focusing on the NPs that show a loss in diameter, there are eight particles that have decreased in diameter more significantly than the others, as shown in Figure 4 and the higher magnification images in Section S8, Figure S8. The majority of the NPs in Figure S8 show a decrease in ADF‐STEM image intensity after AST, as well as in diameter. This is associated with a reduction in the number of Pt atoms scattering the electron beam for each image pixel and indicates that the Pt NP is also reducing in thickness. Conversely, in one case, number 64, whilst the NP has decreased in area, the intensity appears to have increased, suggesting this particle may not be losing atoms but has instead changed shape. For a more detailed understanding, image analysis models would be required to correlate image intensity to volume information (and number of atoms contained within a NP) and not area.

Previous work investigating the stability of Pt at the higher potentials, >1.0 V vs. RHE [8, 12], found that scanning above 1.05 V vs. RHE promoted anodic Pt dissolution. This was postulated to be a transient (and not continuous) process [8], associated with sub‐surface oxide formation and the place exchange of Pt atoms [8, 53]. As the potential is increased further, the dissolution rate decreases as the Pt surface passivates. Subsequent reduction of the Pt oxide was required in order for the native Pt surface to be again revealed [5]. For our experiments, Pt ICP–MS measurements taken after AST cycling and representative of the entire immersed area of the Pt NP BDD‐TEM substrate detected a small (above the background) ∼300 ppt increase in dissolved Pt ions (Section S9). This data, in combination with Figure 3b,c, indicates that during AST cycling, transient Pt dissolution [8, 53] is a likely explanation for the IL‐ADF‐STEM microscopic observation of some of the Pt NPs decreasing very slightly in diameter (less than a single layer of atoms). Dissolution is most likely to occur only during the first few cycles (once a hydrocarbon‐free Pt surface has been exposed) as the lower potential limit of the carbon corrosion AST cycle is not negative enough to promote the reduction of Pt oxide back to Pt. Once the Pt becomes fully passivated, further dissolution is unlikely (at these potentials). For future work, by carrying out IL‐ADF‐STEM during the early cycles of AST, more information can be provided on the timescales over which the observed events, i.e., dissolution, growth, coalescence, atom bridge formation, etc, occur.

Interestingly, TEM data for studies using Pt NPs on CB under similar carbon corrosion cycling conditions showed marked changes, in particular, sizeable increases in average Pt NP size and significant coalescence [5], leading to a loss in activity. This is in direct contrast to the results shown here. Whilst this data highlights the improvements in Pt NP stability that can be made by the employment of the corrosion‐free support electrode BDD, it also emphasizes the requirement for computational investigations. On CB, the Pt NP changes were associated with both a weakening of the Pt─CB bond during oxidation of the surface and NP detachment. Hence, there is a need to explore the differences between the oxygen‐terminated BDD‐Pt surface interaction [64] and that of CB‐Pt, under oxidizing conditions; the well‐defined crystallography of this BDD surface [18, 47] lends itself well to computational modelling. However, the impact of NP size should not be neglected. In the Pt NP‐CB study, slightly larger Pt NPs were employed (2‐4 nm) compared to those used here (1.4–1.6 nm). It is thus important to also ascertain what role Pt NP size plays, in addition to the impact of a changing support. This is due to the fact that smaller Pt NPs are thought to passivate more easily [13], even though initial dissolution rates would be expected to be higher, due to the high surface area to volume ratio.

**FIGURE 5:**
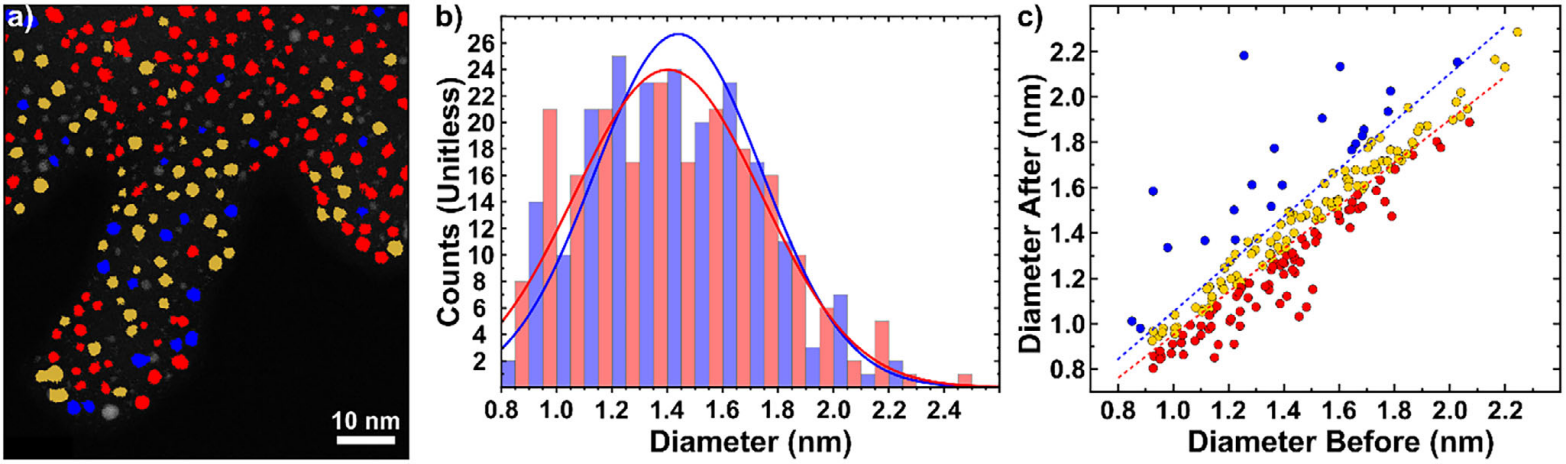
(a) High‐magnification ADF IL‐STEM image with each NP localized, red spots indicate NPs that have decreased in size, blue indicates NPs that have increased in size, and gold indicates NPs that are unchanged. (b) Histogram of NP diameter, blue trace are particles before AST, red trace are particles after AST (c) Scatter plot of NP diameter as a function of initial diameter, with gold spots representing unchanged NPs within ±5% error, blue spots represent those which show a diameter increase, >5% (blue error line), red spots are particles that have decreased in size below the 5% red error line.

To test the robustness of the image analysis model and to see whether the same trends hold in other areas of the BDD‐TEM electrode, two additional areas were analyzed: Figure 5 and Figure S9 in Section S10. Figure 5a shows the resulting particle size changes shown on the “Before AST” BDD‐TEM image. In this area, *n* = 203 NPs were analyzed. The NP diameter distribution histogram (red), Figure 5b gives the same mean NP diameter of 1.4 ± 0.3 nm “Before and After AST”. Figure 5c shows a plot comparing the “Before AST” NP diameter with the “After AST” NP diameter, on a particle‐by‐particle basis, with the ± 5% error lines included. Those NPs that show an increase in diameter (outside the estimated error) are colored blue = 21 out of 203 NPs (10%), whilst those that show a diameter decrease (outside the estimated error) are colored red = 78 out of 203 NPs (38%). For those that have increased significantly in diameter, similar processes are evident, i.e. coalescence, disassembly of surrounding clusters/NP, etc to those observed in Figure 4. Five NPs disappear completely from the “After AST” image. NPs that show a diameter change within the estimated 5% error = 104 out of 203 NPs, 51% are shown as gold circles. Similar to the previous data set, and as shown in Figure S5, Section S5, when plotting the NP area, the same trends are observed, indicating that approximating the particles as circular is again valid. An amalgamated version showing the combined data for all three sets is shown in Section S11, Figure S10, n = 545 NPs.

It is also possible to look for correlations between a NP diameter and its edge to edge nearest neighbor distance, with respect to % change in NP diameter as a result of AST. Interparticle distance is thought to play a critical role in Pt NP stability [13]. The amalgamated data for all three areas is shown in Section S12a, Figure S11. For the range of nearest neighbor distances interrogated from ∼0.1–3.0 nm, changes in NP diameter are observed over all distances for both growing and shrinking particles. The majority of the most significant changes occur when the nearest‐neighbor distances are less than ∼1.0 nm, independent of whether the NP is growing or shrinking. This also highlights the dynamic interplay between very closely spaced NPs. The NP trajectory, the distance each NP travels as a result of AST (data analysis as described for Figure S6 in Section S6), was carried out for the entire data set, n = 545, Section S12b. As Figure S12 shows, the average trajectory length is short, ∼0.36 nm, which rules out significant Pt NP mobility in this system on the oxygen‐terminated BDD support.

## 3 Conclusions

Pt NP (mean diameter ∼1.4–1.6 nm) stability measurements were made using IL‐TEM under AST conditions, which not only assess the start/stop process of a fuel cell but also induce sp^2^ carbon corrosion. Here, a carbon corrosion potential range (1.0–1.6 V vs. RHE) was employed, which has a slightly higher upper limit than the DOE protocol [11]. A BDD (negligible sp^2^ carbon) electrode thinned to electron‐beam transparency to enable IL‐TEM measurements was used for these measurements. Coastline mapping of the edge of the Pt NP coated BDD electrode pre‐ and post‐AST showed no recession. This observation reinforced the corrosion‐free characteristics of the electrode and enabled assessment of Pt NP stability free from convolutions with carbon corrosion. In combination with advanced image analysis, changes to individual Pt NPs on a NP‐by‐NP basis (∼200 NPs per image) were tracked after 1000 AST cycles. NP area (and by inference diameter, assuming circular particles) and center of intensity position for individual NPs before and after AST were analyzed using home‐written scripts. IL‐ADF‐STEM images revealed ∼40–50% of the Pt NPs did not change area, within the estimated error of the measurement. The majority of the remaining NPs underwent a very small decrease in area, thought to be due to a transient (place‐exchange) dissolution process, whilst a limited number increased slightly in area. Higher resolution images also showed the formation of atom bridges between NPs and disassembly of neighboring atom clusters. This data contrasts with observations made using TEM for Pt NPs on CB (under similar cycling conditions) where sizeable increases in NP size were observed, and significant coalescence noted [5]. This points to a more detailed understanding of the Pt‐BDD surface interaction being required and further highlights the potential of BDD as a catalyst support. With advances in BDD particle synthesis methodologies [65], BDD particulate electrodes, for use by the community, become a realistic future possibility. Our observed changes would also be impossible to discern using methodologies that sum all events across an electrode surface, such as analysis of the H_ads_/H_des_ region of a CV or ICP‐MS of dissolved Pt. It would be of interest to use this platform to further explore the timescale over which the observed changes occur, the impact of Pt NP size and spacing, different potential cycling conditions (for example, the AST cycle, which targets Pt dissolution/degradation [13]), and the evaluation of electrocatalytic NP systems where carbon corrosion is problematic [66].

By moving to higher magnification and through the use of the TEM intensity data associated with individual NPs, it will be possible to advance image analysis further by both counting the number of atoms per NP and reconstructing the 3D shape of the NP [67]. This will enable changes to each NP in terms of the number of atoms lost/gained to be quantified and allow distinctions to be drawn between treating the NPs as an area vs. a volume. This level of detail should help facilitate understanding of why some NPs of a similar area favor stability over transient dissolution and reveal the atom‐scale mechanisms by which NPs grow and dissolve.

## 4 Experimental

### 4.1 Solutions and Materials

Solutions were made using <18.2 MΩ cm ultra‐pure deionized water (Milli‐Q, Millipore). For AST, 0.5 M perchloric acid (70%, 99.99% trace metals basis, Sigma–Aldrich) was employed. BDD was provided by Element Six, Oxford, UK, and was grown using microwave chemical vapor deposition under conditions that promote negligible sp^2^ carbon content (Electroanalysis grade BDD, material E in reference [68]). The BDD was grown thick enough (>0.4 mm) in order to be removed from the non‐diamond growth support. The material was suitably doped with boron, ∼3 × 10^20^ B atoms cm^−3^, such that the material was above the metallic threshold [68]. Both surfaces were lapped and then polished on a resin‐bonded wheel to thin the material to <80 µm with ∼nm surface roughness. Laser micromachining of the BDD was carried out using a 355 nm Nd:YAG 34 ns laser (Oxford Lasers) to cut out disks, 3 mm in diameter. To remove machining debris, the electrodes were acid cleaned by immersing them in concentrated H_2_SO_4_ (5%–97%, Scientific and Chemical Supplies Ltd.), saturated with KNO_3_ (99.0%, Scientific and Chemical Supplies Ltd.) at ∼200°C, for 30 min, followed by rinsing with ultra‐pure water before cleaning for 30 min in concentrated H_2_SO_4_, at ∼200°C [18, 69]. The rounds were Ar ion milled (GATAN PIPS II) to produce electron beam transparent TEM substrates of ∼nm surface roughness. This process is described in detail in reference [18]. For electrical contacting, the BDD TEM substrate was masked with a glass slide, leaving a small segment of the disk exposed (∼0.5 mm from the edge). This was then laser roughened (532 nm Nd:YAG 15 ns laser), which also converts the top surface of this region into amorphous sp^2^ carbon [69]. Further acid cleaning was undertaken to remove any machining debris. A carbon ink (MG Chemicals, 838AR) electrical contact was painted onto this roughened area.

Pt NPs were sputter‐coated onto the BDD TEM substrate using a benchtop sputter coater (SC7640, Quorum Technologies) with an accelerating voltage of 2 kV for 3 s under vacuum. The contact was masked using a glass slide.

### 4.2 Electrochemical Measurements

Electrochemical experiments on a BDD TEM grid were carried out using the set‐up outlined previously [18]; a schematic is provided in Section S1. Stainless steel tweezers were used to contact the BDD‐TEM substrate (and allow easy manipulation) and provide an “electrical wire” for connection to the potentiostat via a crocodile clip. The BDD TEM substrate was dipped into the solution of interest, such that the central hole was covered, but avoiding solution touching the C ink contact. For BDD‐TEM experiments, an Ag|AgCl reference electrode (non‐leak DRIREF, ∼3.5 M KCl, WPI) and a high surface area Pt coil counter electrode were employed, surrounding the working electrode. For AST, the potential was cycled at a scan rate of 0.1 V s^−1^ in a potential window of 0.80–1.40 V vs. Ag|AgCl (1.0–1.6 V vs. RHE) in 0.5 M perchloric acid. The open circuit potential was ∼0.68 V vs. RHE before AST, rising by ∼0.25 V after AST. The pH of the solution was measured as 0.30 (pH meter, Seven Multi, Mettler Toledo, UK). Cyclic voltammetry was carried out using an Ivium CompactStat Standard potentiostat (Eindhoven, Netherlands).

### 4.3 TEM Imaging

For IL‐TEM imaging experiments, the coastline around the hole‐edge of the BDD‐TEM electrode was first imaged using a double‐corrected JEOL JEM‐ARM 200F TEM, operated at 200 kV, using an ADF detector with a convergence semiangle of ∼25 mrad, a detector inner angle of ∼50 mrad and a probe size of ∼0.06 nm. Three areas of typical size 70 × 70 nm were selected, and ADF‐STEM images were taken in the same areas prior to and after AST, Section S2. For  IL‐TEM imaging, it is important to ensure the physical alignment (orientation of the grid and any tilt on the sample) of the BDD TEM disk is as close to identical as possible. This simplifies both finding the identical locations and image analysis.

Upon removal from electrolyte solution, the BDD‐TEM substrates were gently stirred in ultra‐pure water for ∼30 s to remove any salt residue and then baked under high vacuum (10^−5^–10^−6^ torr) at 60°C for ∼12 h (overnight). The latter was to reduce hydrocarbon build‐up, which can contaminate the surface under the electron beam. 60°C has been shown previously not to impact Au atom/NP size and position on a BDD TEM substrate [60].

### 4.4 Image Analysis

**FIGURE 6:**
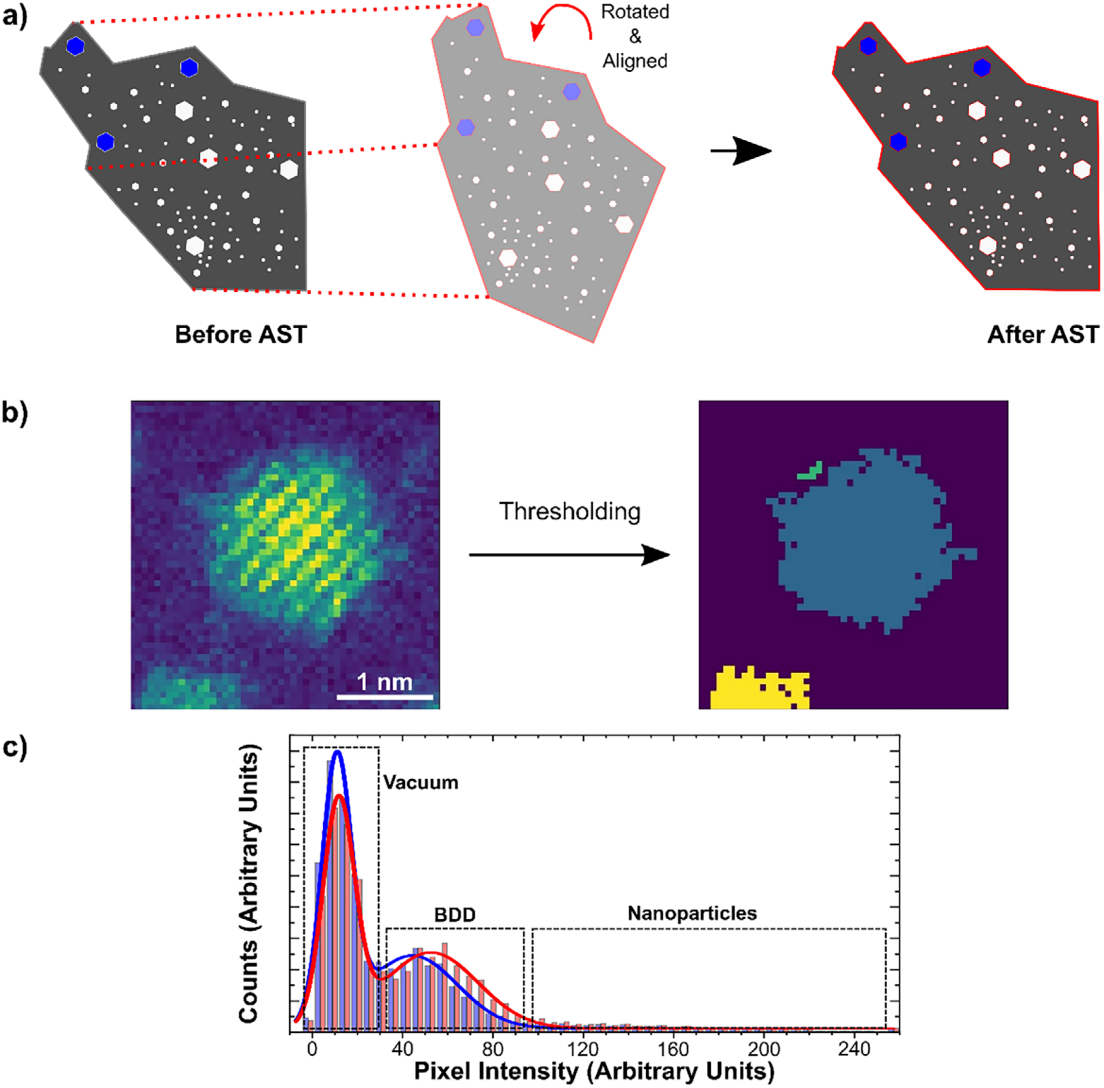
Image processing steps for IL‐TEM Pt NP analysis. (a) Rotation of the “After AST” image to match the orientation of the “Before AST” image. (b) Each NP located in (a) is cropped to a 50 × 50‐pixel image, with the NP forming the center of the image. The BDD background is removed using a threshold obtained by doubling the mean BDD pixel intensity from the data in (c); the data in (b) is real data.

“Before AST” is the image acquired before AST; “After AST” is the image acquired after AST (in the same location). The first step in the image analysis is alignment of the “Before AST” and “After AST” images (Figure 6a). Each hole in the BDD‐TEM substrate has a distinctive “jagged” edge. The dark region in Figure 6a represents the BDD‐TEM substrate, the white region the vacuum (the hole region), and the interface between the two is the edge. Whilst every best effort is made to place the BDD‐TEM electrode in the TEM holder with exactly the same orientation for IL‐TEM, this is challenging. Thus, a small rotation error (usually a few degrees) can be introduced into the “After AST” image. To correct for this, the “After AST” image is rotated (and if necessary translated) so that the BDD hole edges overlap, using a transformation matrix generated using the OpenCV library in Python (Version 4.9.0) [70]. This process also makes use of “anchoring” Pt NPs (blue in Figure 6a), NPs that undergo minimal changes in size (<0.1%) and position (< image pixels) during AST (Figure 6a, blue particles).

To locate the center position (*x*,*y*), referred to as the center of intensity, for each NP, the trackpy library (Version 0.62) [71] is used, which is an implementation of the Crocker–Grier method [35, 72], which treats the NPs as 2D Gaussian functions. This is a fair assumption, as the vast majority of NPs always have a higher TEM pixel intensity at the center than at the edges. After the particles have been located and trajectories measured, assessment of changes in the size (pixel area) and diameter of individual NPs is made. For each NP in both the “Before AST” and “After AST” images, the images are cropped such that each NP has its own 50 × 50‐pixel image (Figure 6b, left). One pixel at the resolution of the recorded images is 0.064 nm × 0.064 nm, which is ca. four times less than the atomic diameter of a Pt atom = 0.27 nm [49]. Thresholding is then applied to isolate the Pt NP from the BDD TEM background.

To determine the correct threshold value to use, pixel intensity histograms, containing contributions from the vacuum, BDD and Pt NPs (Figure 6c) across the entirety of the “Before AST” (blue) and “After AST” images (red), are analyzed. These histograms are fitted using two Gaussian functions for the dominant vacuum and BDD responses Figure 6c. A threshold for each NP image in the “Before AST” and “After AST” TEM images is set at twice the mean pixel intensity of the background BDD signal. For this threshold value, 96% of the BDD pixels are excluded without compromising Pt pixels. Residual background pixels as well as holes in the Pt‐covered regions are removed using the scikit‐image library (Version 0.23.0) [73]. This library is also used to isolate the Pt NP, which is located in the center of the image, as shown in Figure 6b for the NP of interest highlighted in blue (“After AST ”). Finally, NP areas can be extracted using param_extraction, a homemade function based again on functions of the same library.

## Conflicts of Interest

The authors declare no conflicts of interest.

## Supporting information




**Supporting File**: smll74547‐sup‐0001‐SuppMat.pdf.

## Data Availability

All raw data used in the preparation of this manuscript can be found at the Warwick Research Archive Portal http://wrap.warwick.ac.uk/194325/.
