# Supplementary material for: Evaluating Pt Nanoparticle Electrocatalyst Stability on Corrosion Free Carbon Supports Using Identical Location TEM
Source: Small. 2026 Jul 24;22(50):e13549. doi: 10.1002/smll.202513549 (PMC13548911; doi:10.1002/smll.202513549)
Supplement: Supplementary file 1 [file SMLL-22-e13549-s001.pdf]

Evaluating Pt Nanoparticle Electrocatalyst Stability on Corrosion Free Carbon Supports Using Identical-Location TEM

Daniel Houghton[1], Yisong Han[2], Richard Beanland[2], Louis Godeffroy[3], Frédéric Kanoufi[3], Viacheslav Shkirskiy[3], Jonathan Sharman[4], Julie V. Macpherson[1]

[1]Department of Chemistry, University of Warwick, Coventry, CV4 7AL, UK,

[2]Department of Physics, University of Warwick, Coventry, CV4 7AL, UK,

[3]Université Paris Cité, CNRS, ITODYS, 75013 Paris, France,

[4]Johnson Matthey Technology Centre, Reading, RG4 9NH

SI 1: Schematic of Experimental Set-up

SI 2: Three IL-ADF-STEM Pre- and Post-AST Electrochemical Cycling Images

SI 3: Electrochemical assessment of the H_{ads} / H_{des} potential region of the Pt NP – BDD TEM electrode

SI 4: Area Error Calculation

SI 5: Image Analysis by Area

SI 6: Electron Beam Induced Movement

SI 7: Growing Particles Data Set 1

SI 8: Shrinking Particles Data Set 1

SI 9: ICP – MS

SI 10: Third IL-ADF-STEM Data Set

SI 11: Combined Data Set Showing All Particle Size Changes

SI 12: (a) Edge to Edge Nearest Neighbours and Impact on NP Diameter Changes: (b) NP trajectories

References

SI 1: Schematic of Experimental Set-up

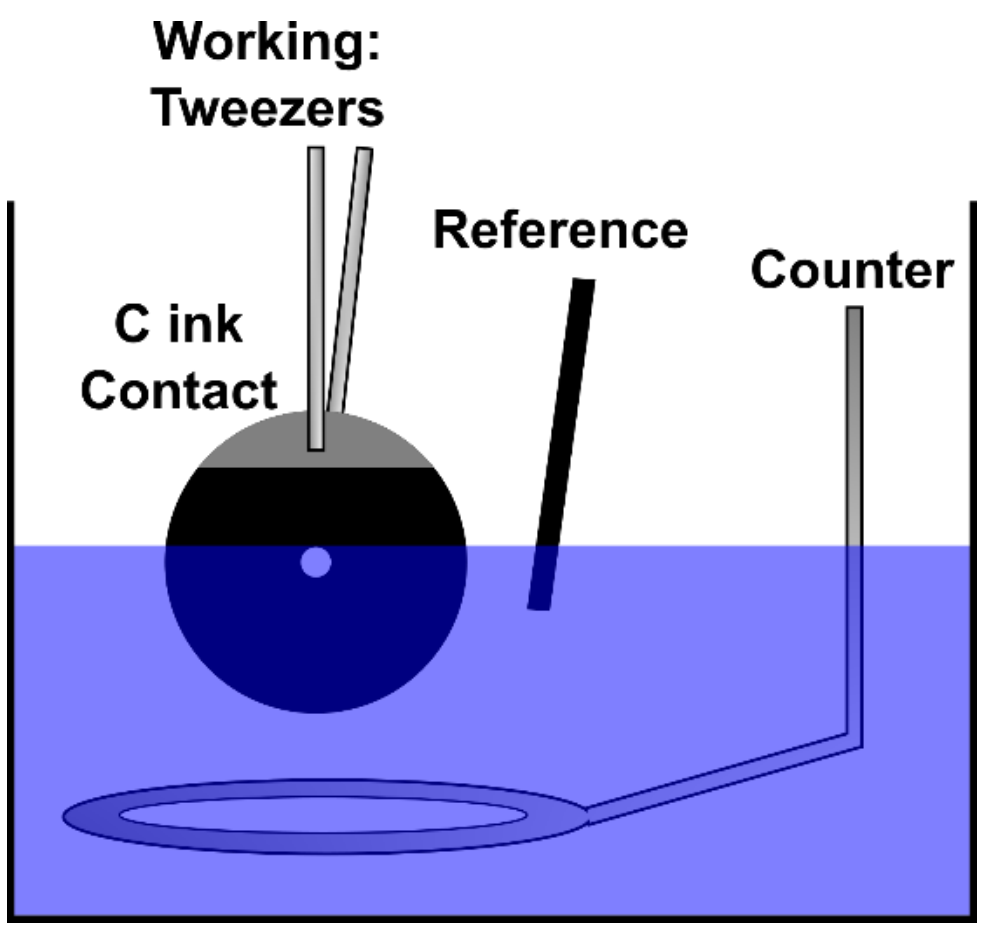

Figure S1: Schematic of the experimental set-up employed. The working electrode is a BDD TEM electrode,[1] the reference is a RHE or 3.5 M Ag|AgCl electrode and a high surface area Pt ring serves as the counter electrode. Metal tweezers make a contact with the BDD-TEM electrode in the region of the C ink electrical contact, which is excluded from solution.

SI 2: Three IL-ADF-STEM Pre- and Post-AST Electrochemical Cycling Images

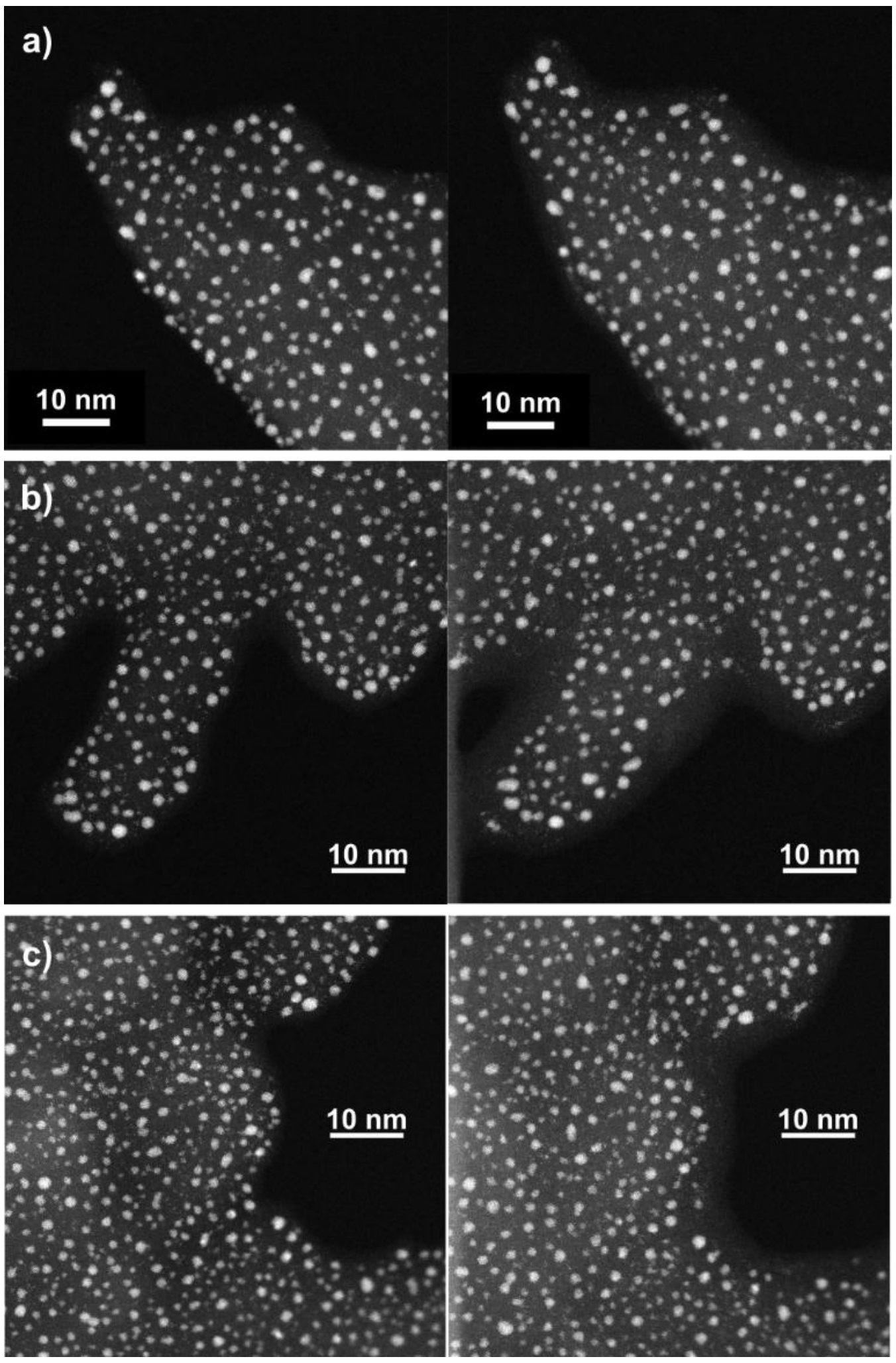

Figure S2. The three IL-ADF-STEM image pairs recorded; left “Before AST” and right “After AST”. (a) and (b) are analysed in the main text of the paper.

SI 3: Electrochemical assessment of the H_{ads} / H_{des} potential region of the Pt NP – BDD electrode

For CV analysis of the Pt NPs in acid, measurements were carried out using a Vionic potentiostat (AutoLab, Metrohm UK) in true linear (analogue) mode.[2] Moving away from a digital (staircase) approach is important when accurate electrochemical assessment of surface-bound processes is required, *i.e.* H_{ads}/H_{des}.[2,3] For these measurements a 3 mm diameter BDD macroelectrode (of the same material used to make the BDD-TEM electrodes), sealed in Rigid10k (PMMA/Glass, FormLabs) was used alongside an RHE (Mini-HydroFlex, Gaskatel, Germany) reference electrode and a high surface area Pt coil counter. Experimental conditions were deaerated 0.5 M perchloric acid with the potential scanned reductively from 0.6 V to 0.02 V *vs* RHE and back at 100 mVs^{-1} in the H_{ads} / H_{des} region.[2,3] Figure S3 shows the true linear CV response for both the "Before" and "After" AST surfaces scanned CVs.

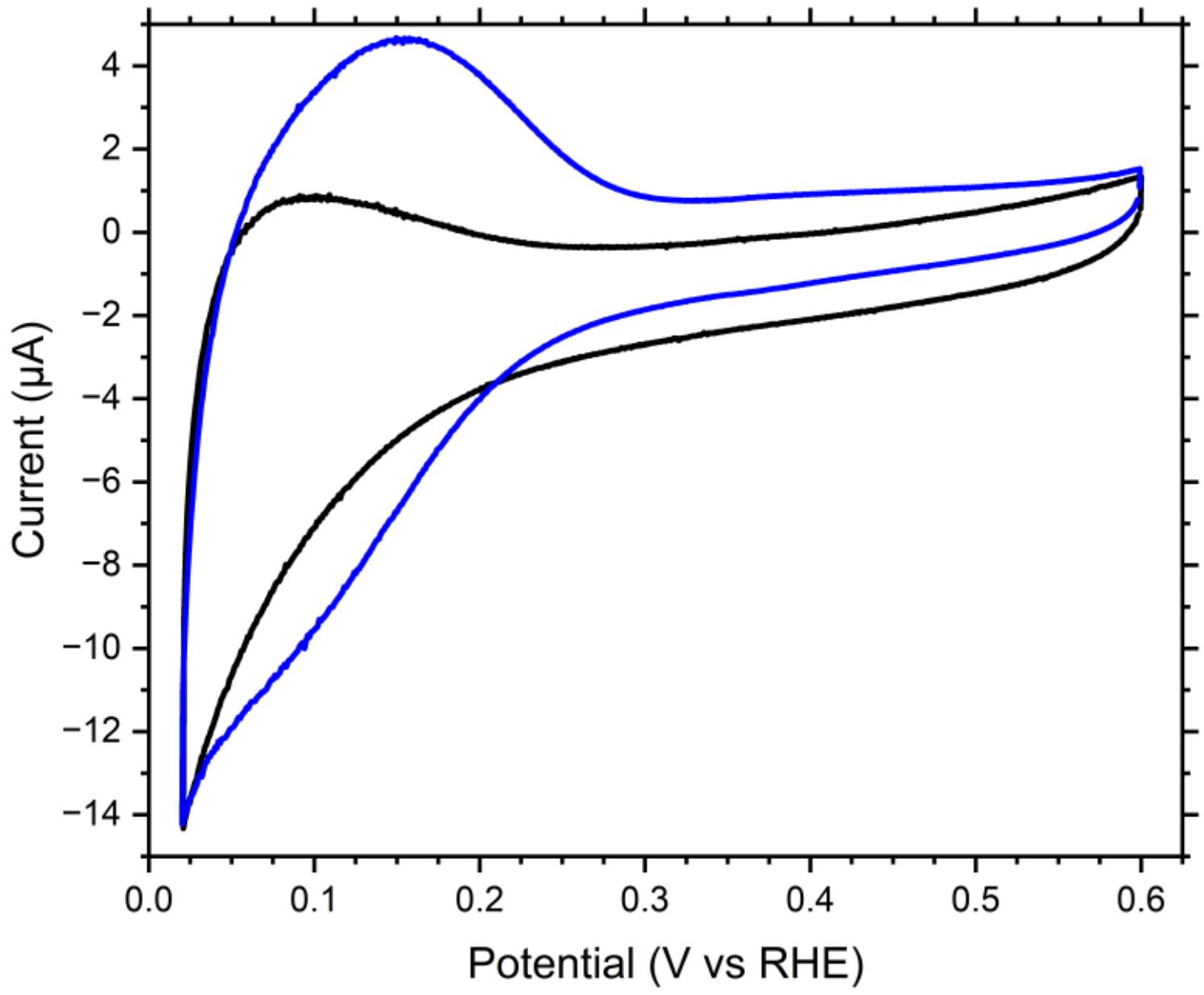

Figure S3. True linear (analogue) second scan CV for the sputtered Pt NPs "Before AST" (black line) and "After AST" (blue line) on a 3 mm diameter BDD electrode in a deaerated solution containing 0.5 M perchloric acid at 100 mV s^{-1}. The CV starts from 0.6 V *vs* RHE and scans reductively to 0.02 V and back.

In the region 0.0 to 0.3 V *vs* RHE, hydrogen adsorption (H_{ads}) and hydrogen desorption (H_{des}) signatures should be visible, which scale with Pt surface area and show a dependency on crystal facet.[4] The black line data in Figure S3 shows the second scan CV data for the as-sputtered Pt NPs on BDD. No significant H_{ads}/H_{des} signatures are visible, which likely suggests adsorbate

contamination of the surface.[5] The blue line data, "After AST" has H_{ads} / H_{des} region which indicates an increased (~ ×1.5) electrochemical surface area compared to the as-sputtered surface. This data supports removal of the contaminant. The AST may also act as a form of "pre-conditioning" resulting in formation of a stable re-organized surface on which reproducible measurements can be conducted.[5]

SI 4: Area Error Calculation

A ± 5% overall measurement error in the NP radius is assumed: This is derived from the error in the BDD pixel intensity peak position (± 6%; derived from 1 standard deviation from fitting the BDD intensity with a Gaussian function) for the BDD substrate. This is likely to be the largest source of error in the experiment, as an error in the threshold will propagate throughout the entire analysis. Feeding this value (±6% of the threshold) into the thresholding component of the image analysis produces a maximum of ±10% difference in the final measured area of the NP. This equates to a percentage error in a calculated NP diameter of 5%.

Area, A, of a circular NP of radius r is given by equation S1

$$A = \pi r^2 \qquad \text{(S1)}$$

$$\frac{dA}{dr} = 2\pi r$$

$$dA = 2\pi r dr$$

$$\frac{dA}{A} = \frac{2\pi r dr}{A} = \frac{2\pi r dr}{\pi r^2} = \frac{\mathbf{2} dr}{r}$$

"% Error in A" = $\frac{dA}{A}$ [= $\frac{\mathbf{2} dr}{r}$]

"% Error in r" = $\frac{dr}{r}$

∴ % error in A = 2 × % error in r

∴ 10% (measured) error in A = 5% error in r (calculated)

SI 5: Image Analysis by Area

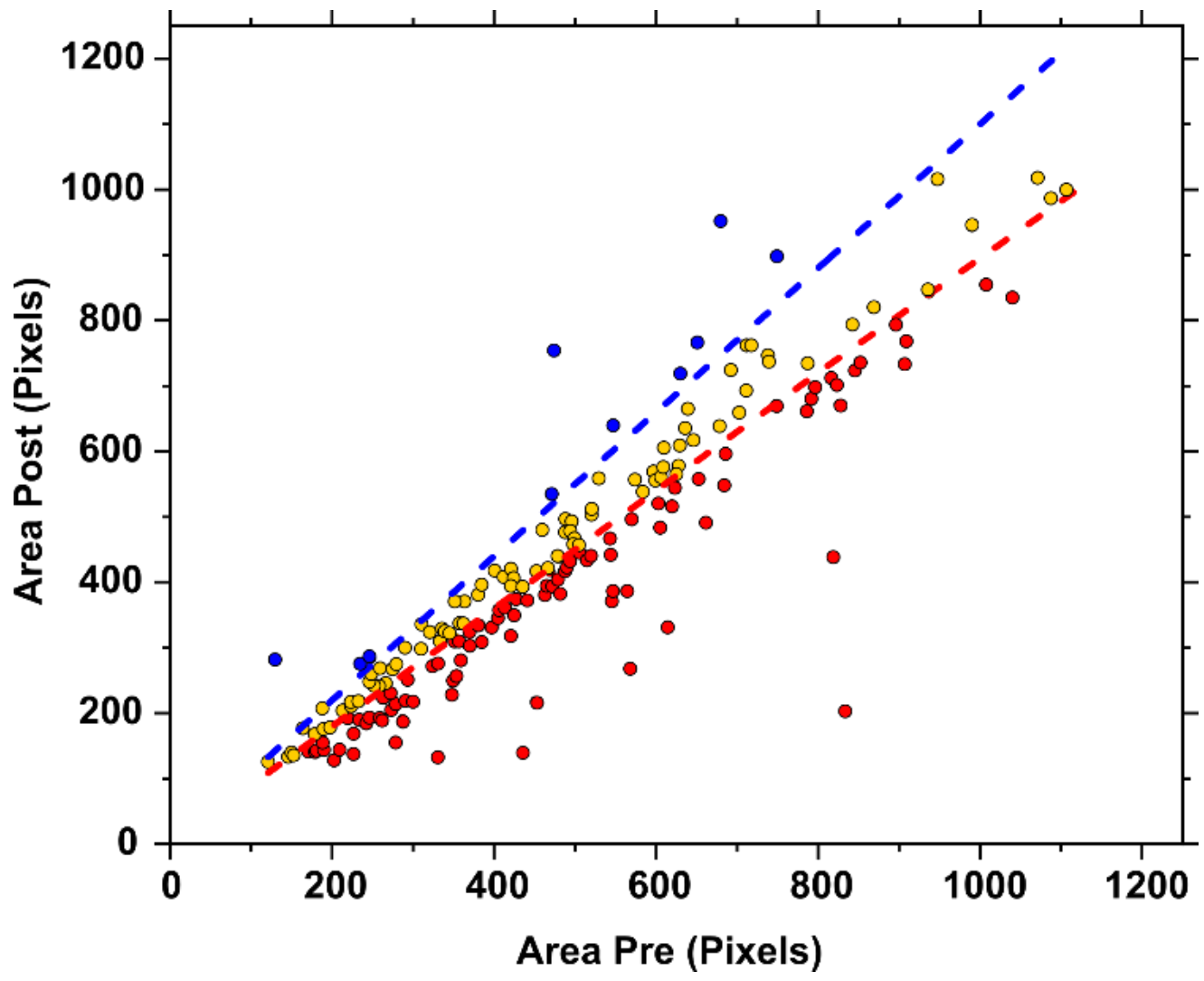

Figure S4. For the first data set presented in the main text (Figures 2,3), here a 10% error in area is plotted (blue = +10%, red dash = -10%). NPs that have decreased in area are red, NPs that have increased in area are blue and NPs that are “unchanged” (within error) are gold. Note, as detailed in **SI 4**, a 10% error in NP area corresponds to a 5% error in NP diameter.

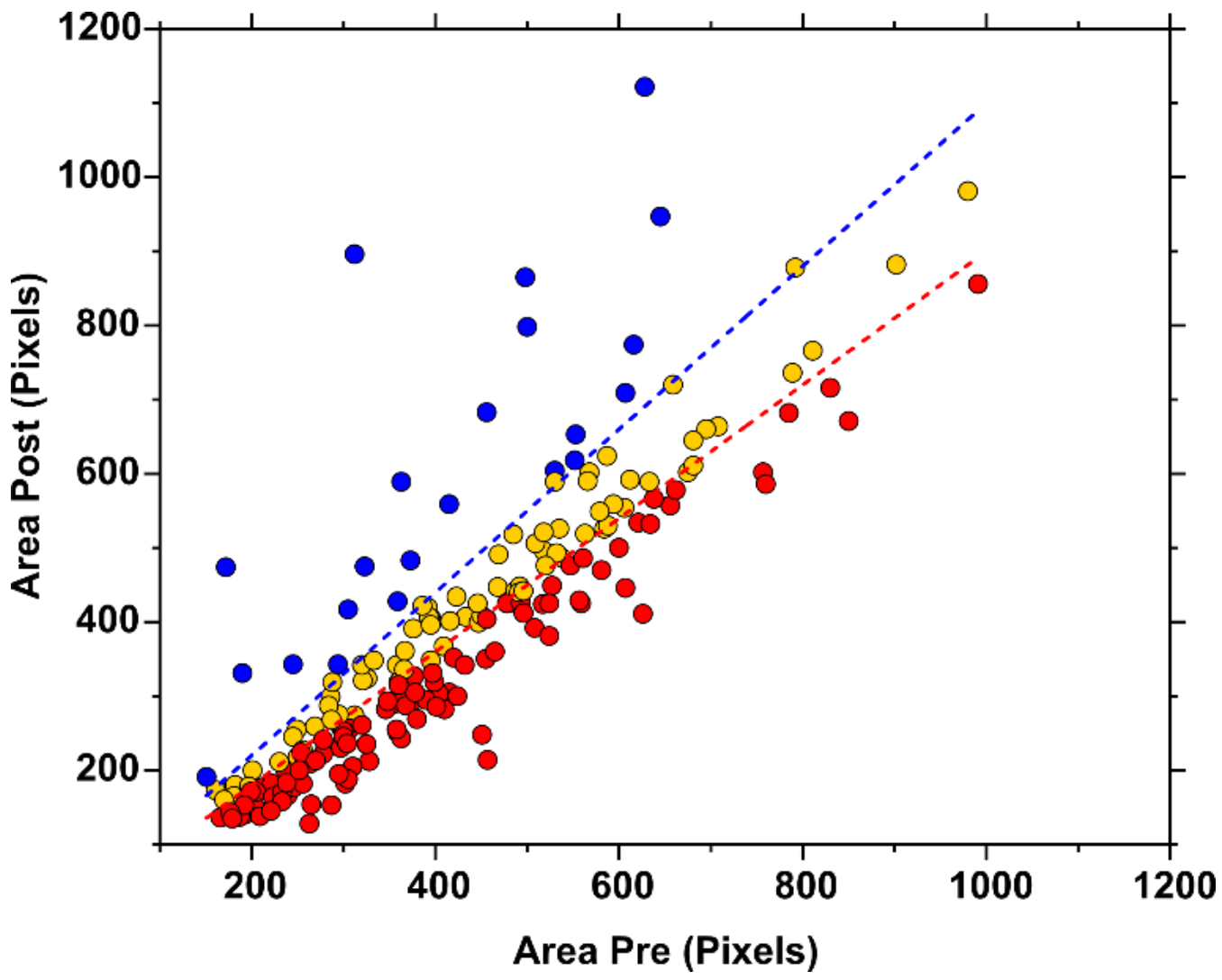

Figure S5. For the second data set presented in the main text (Figure 5), here a 10% error in area is plotted (blue = +10%, red dash = -10%). NPs that have decreased in area are red, NPs that have increased in area are blue and NPs that are “unchanged” (within error) are gold.

SI 6: Investigation of Electron Beam Induced Movement of Pt NPs on the BDD surface

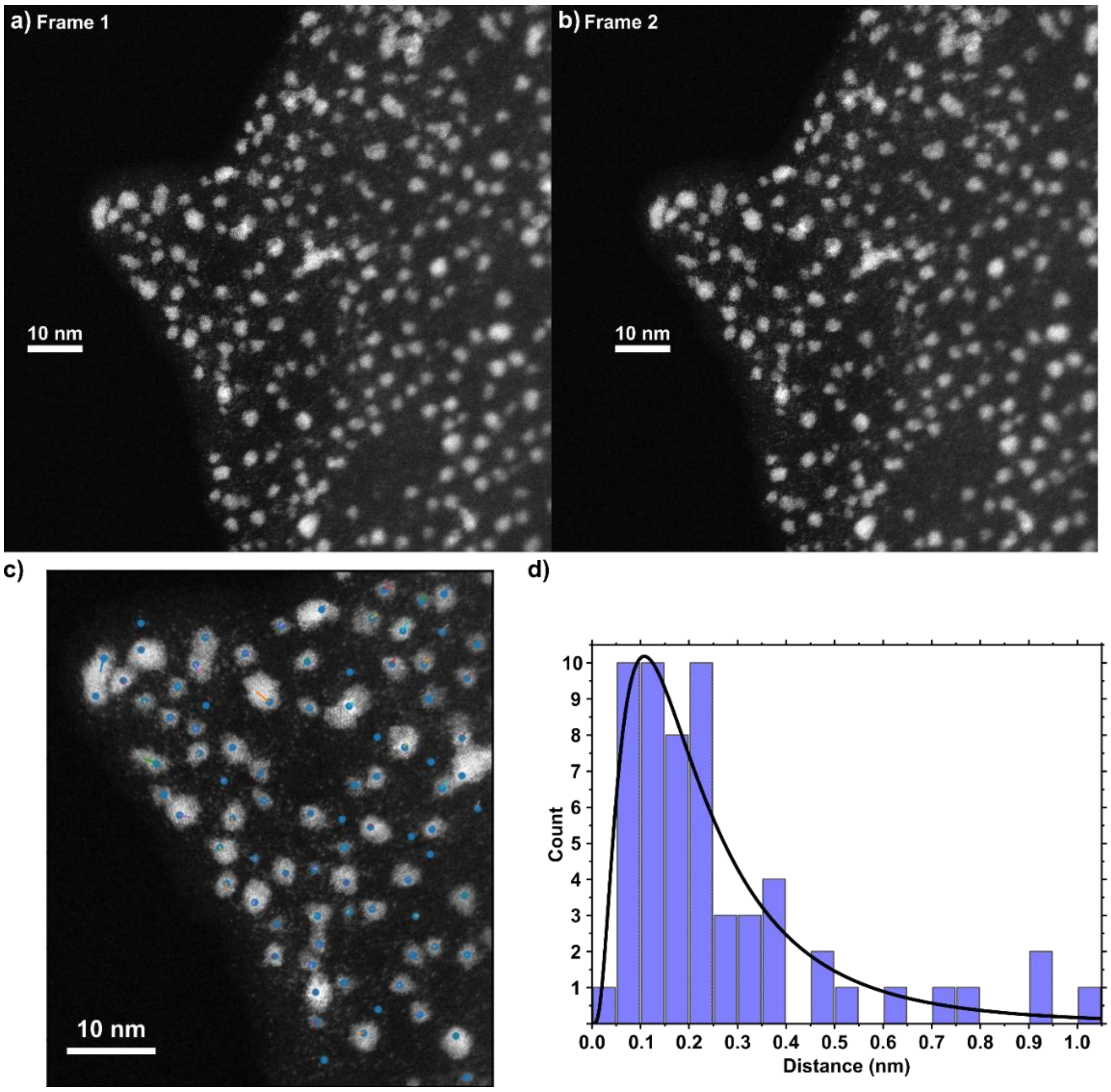

Figure S6: (a, b) Two consecutive IL-ADF-STEM frames are taken with the same TEM imaging conditions the same as for the data sets presented in the main text. (c) The second frame after image analysis. Blue spots highlight a NP's initial centre of mass with trajectory lines drawn to show the NP's final centre of mass. (d) Trajectory distance versus nanoparticle number, histogram for NPs in (c).

SI 7: NPs which grow in size during AST

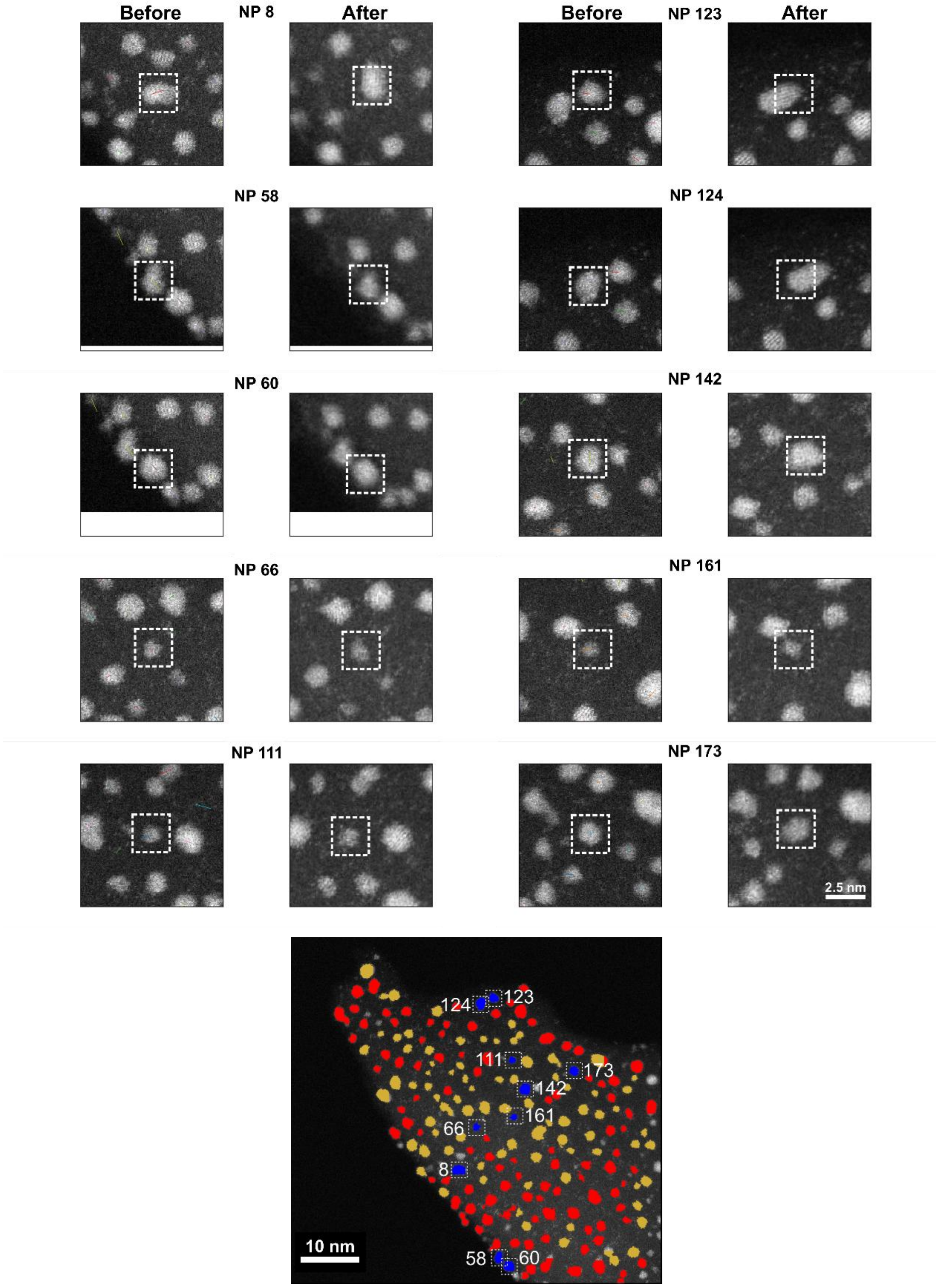

Figure S7: All NPs that have grown during AST are highlighted in blue in the IL-ADF-STEM "Before AST" image (bottom of image). Each blue NP is highlighted with a white box. The size of each zoomed in "Before" and "After" AST image is 9.6 × 9.6 nm (150 × 150 pixels).

SI 8: NPs which reduce in size during AST

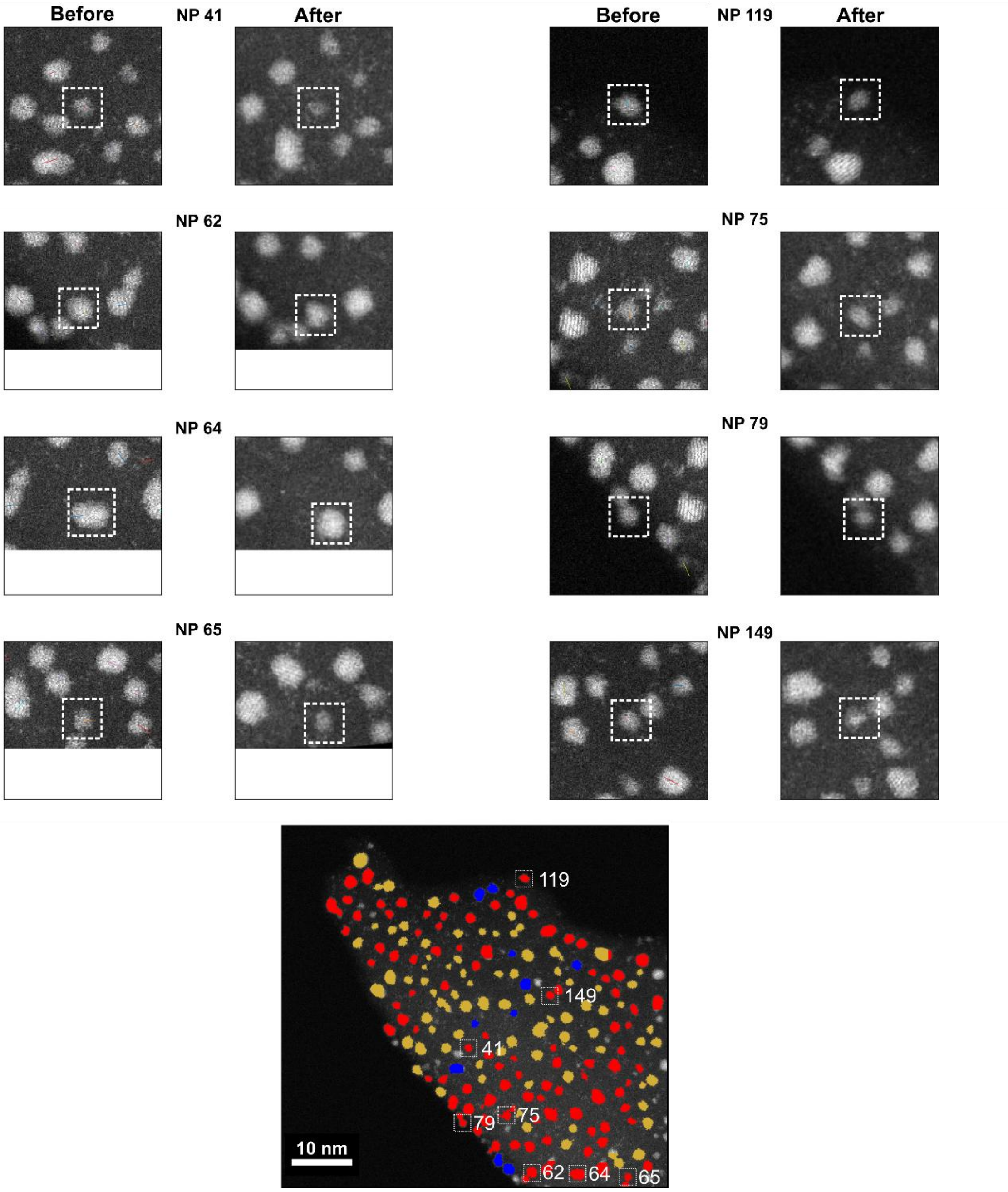

Figure S8: NPs that show a notable decrease in diameter during AST are highlighted in red in the IL-ADF-STEM "Before AST" image (bottom of image). The red NPs which show the largest decrease in size are highlighted with a white box. The size of each zoomed in "Before" and "After" AST image is 9.6 × 9.6 nm (150 × 150 pixels).

SI 9: Pt ICP – MS

ICP – MS Measurements: An Agilent 7900, equipped with SPS4 Auto-sampler was used, with an internal standard (Er 1ppb) and plasma conditions of: plasma gas: 15 L/min, carrier gas: 0.9 L/min, Nebulizer gas: 1.05 L/min, Auxiliary gas: 0.9 L/min, Spray chamber temperature: 15°C, Helium mode gas flow 4.0 ml/min, Forward power 1550W, flow rate: 0.1rps, detector analogue HV 2152V, pulse HV 956V, Discriminator 5 mV. The instrument was auto-tuned before sequence and the P/A factor adjusted using calibration standards during analysis. The instrument was calibrated with Pt standards in a matching background in the concentration range 0-1000 ppb. The detection limit was determined as 0.0279 ppb Pt. ICP – MS was carried out by taking 3 measurements with a 5% tolerance in the result on 10 ml aliquots of the following solutions: (1) A "blank" solution containing 0.5 M perchloric acid and (2) the "After AST" solution.

**Sample**	**Composition**	**Concentration (Pt) / ppb**
Blank 1	0.5 M $HClO_4$ / H_2O, **pre** AST	0.365 ± 5%
DH-Pt	0.5 M $HClO_4$ / H_2O, **post** AST	0.664 ± 5%

Table S1: Pt ICP – MS results from 0.5 M perchloric acid in the electrochemical cell before AST and the same solution after AST. Each result listed is the average of three measurements with < 5% difference between the three taken. Limit of detection is 0.02 ppb.

There is an increase of Pt concentration of ~0.3 ppb = 300 ppt.

SI 10: 3rd IL-ADF-STEM Data Set

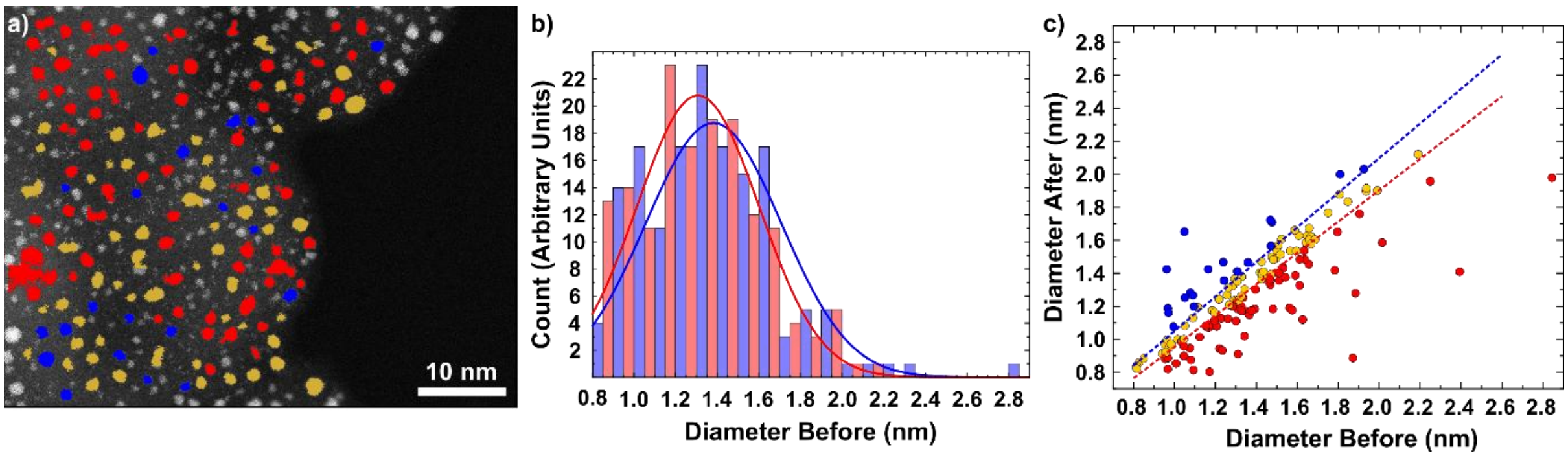

Figure S9: (a) ADF IL-STEM image with tracked NPs highlighted, red indicates a diameter decrease and blue represents a diameter increase outside the estimated error (±5%), gold are unchanged (within the error). (b) Histogram of NP diameter before AST (blue) and after AST (red). (c) Scatter plot of NP diameter after AST as a function of initial NP diameter with the blue circles (solid) representing NPs that have increased in diameter greater than the blue 5% error line, red solid circles, NPs that have decreased in diameter below the red 5% error line, and gold solid circles are unchanged within error.

In this third set of data analysed the average NP diameter before AST was 1.4 ± 0.3 nm, and after AST was also 1.4 ± 0.3 nm. 19 of 153 particles (12%) increased diameter by greater than 5%. 57 of 153 (37%) were "within error" the same and 77 of 153 (51%) decreased in size. The NPs that appear untracked in this image have been filtered out either due to their diameter being below 0.8 nm, or because they are slightly too close to the arbitrary edge boundary (e.g. the particles on the left, top and bottom edges of Figure S9a).

SI 11: Combined Data Set Showing All Particle Size Changes

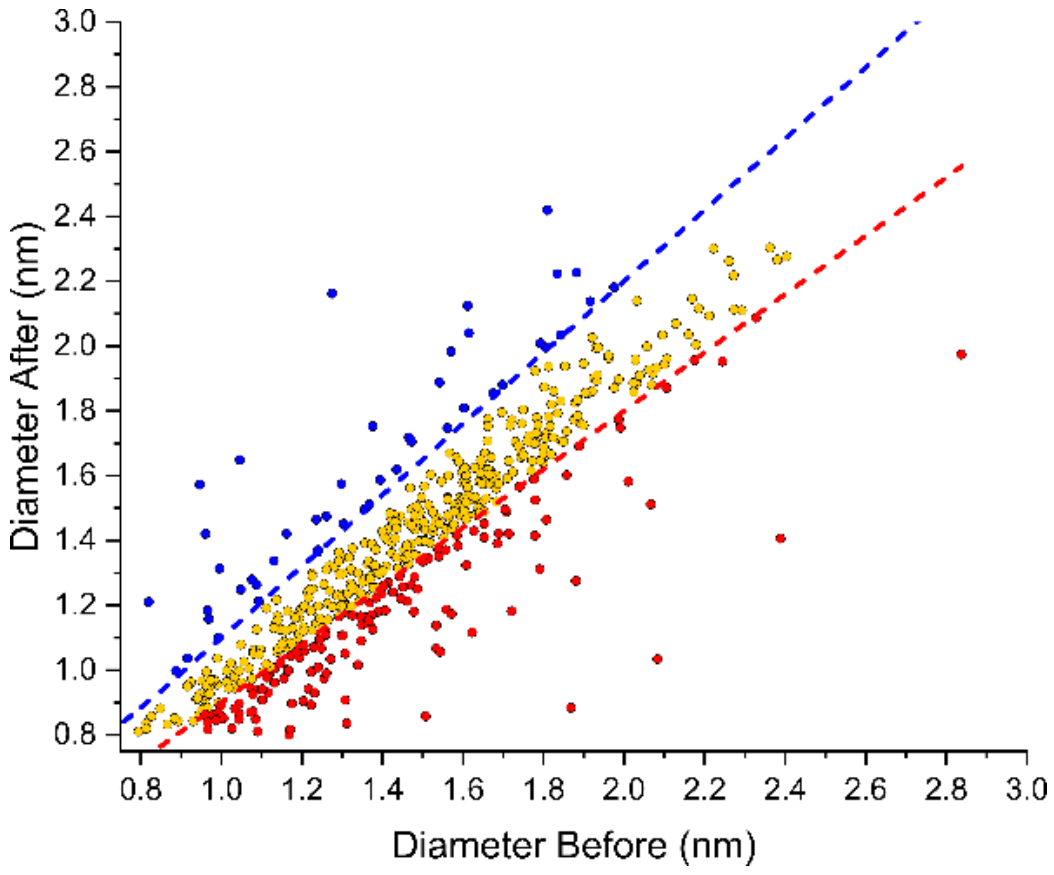

Figure S10: Combined data set of n = 545 NPs from 3 different areas (Figures 3, 5 and S9). Plot of "Before AST" Pt NP diameter versus "After AST" NP diameter on a particle-by-particle basis. Red solid circles indicate NPs which have decreased in diameter, blue solid circles are NPs which have increased in diameter and gold solid circles are NPs that are nominally "unchanged" within ± 5% measurement error, annotated with a +5% blue dash line and a −5% red dash line.

Of all the NPs analysed, 286 / 545 (52%) have decreased in area, 58 / 545 NPs (11%) have grown and the remainder remain unchanged, within the error margin of ± 5%.

SI 12a: Edge to Edge Nearest Neighbours and Impact on NP diameter change

Our analysis procedure enables us to pick a central particle, evaluate the distance of the closest neighbour (edge-to-edge nearest neighbour distance) and then relate this NN distance to whether the central particle diameter has grown or shrunk (or stayed the same) after AST. Figure S11 shows the edge to nearest neighbour distance as a function of change in diameter of the NP.

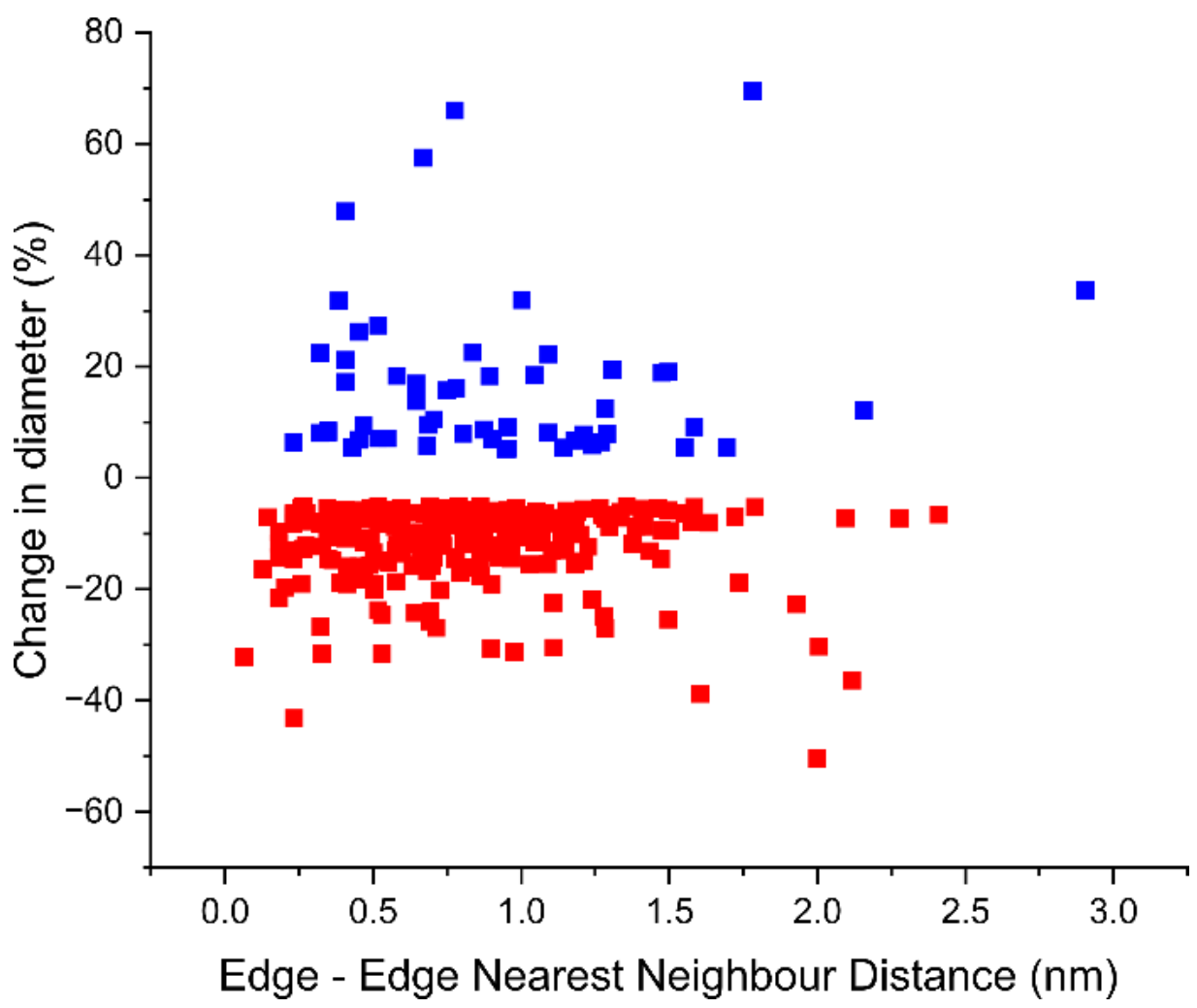

Figure S11: NP edge-to-edge nearest neighbour distance as a function of change in diameter. Particles with diameter changes less than 5% (within error) have been omitted. Red particles have shrunk by >5% and blue particles have grown >5%.

This data set shows two things of interest. Firstly, there is no nearest neighbour cut-off distance for growth or shrinking, i.e. no distance beyond which growth or shrinking events are not observed. This is reasonable considering all nearest neighbour distances are < 3.0 nm in these data i.e. all particles are quite closely spaced.

Secondly: for both shrinking and growing data, the data sets become more clustered, and changes become more significant as the nearest neighbour distance decreases; although we do note a spike at ca. 2 nm. This suggests the magnitude of growth (or shrinking) events is influenced by how close the particles are, with closer distances promoting more dramatic changes in NP size. This aligns with the observation of Ostwald ripening events (since closer particles can facilitate the transfer of atoms between them), however to be fully confident atom counting methods should be used.

SI 12b: NP trajectories

Each NP in the data sets have an associated trajectory, as shown by the green arrows in Figure S12a. The trajectory length histogram of all 545 trajectories is plotted in Figure S12b. The average trajectory length was 0.36 ± 0.23 nm and the directions appear to be random. The very short trajectory length indicates the particles are not especially mobile during this experiment, reducing the chances of aggregation / coalescence events. It is also indicative (in general) of NP stability on the substrate, since if the NP – support interaction was weak, the NPs would be expected to be mobile.

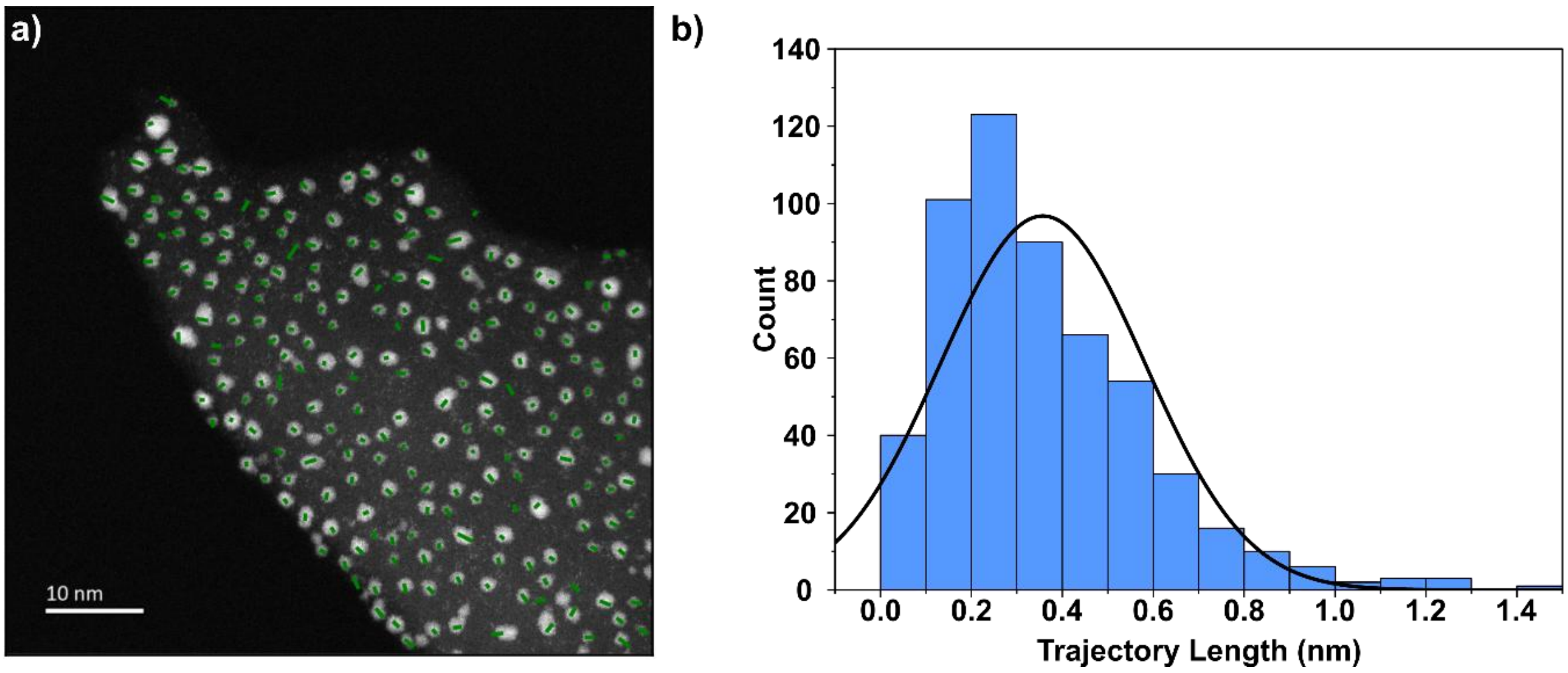

Figure S12(a): Nanoparticle trajectories in the first data set (main text Figure 3) plotted on the Before AST image. Each trajectory is plotted from the centre of intensity of the particle to the new position after AST. (b) Trajectory length histogram for all 545 particles tracked over all 3 data sets.

References

(1) Hussein, H. E. M.; Wood, G.; Houghton, D.; Walker, M.; Han, Y.; Zhao, P.; Beanland, R.; Macpherson, J. V. Electron Beam Transparent Boron Doped Diamond Electrodes for Combined Electrochemistry─Transmission Electron Microscopy. *ACS Measurement Science Au* **2022**, *2* (5), 439–448. https://doi.org/10.1021/acsmeasuresciau.2c00027.

(2) Colburn, A. W.; Levey, K. J.; O'Hare, D.; Macpherson, J. V. Lifting the Lid on the Potentiostat: A Beginner's Guide to Understanding Electrochemical Circuitry and Practical Operation. *Physical Chemistry Chemical Physics* **2021**, *23* (14), 8100–8117. https://doi.org/10.1039/d1cp00661d.

(3) Hai, B.; Tolmachev, Y. V.; Loparo, K. A.; Zanelli, C.; Scherson, D. Cyclic versus Staircase Voltammetry in Electrocatalysis: Theoretical Aspects. *J. Electrochem. Soc.* **2011**, *158* (2), F15. https://doi.org/10.1149/1.3512914.

(4) Zalitis, C. M.; Kucernak, A. R.; Sharman, J.; Wright, E. Design Principles for Platinum Nanoparticles Catalysing Electrochemical Hydrogen Evolution and Oxidation Reactions: Edges Are Much More Active than Facets. *J. Mater. Chem. A Mater.* **2017**, *5* (44), 23328–23338. https://doi.org/10.1039/c7ta05543a.

(5) Shinozaki, K.; Zack, J. W.; Richards, R. M.; Pivovar, B. S.; Kocha, S. S. Oxygen Reduction Reaction Measurements on Platinum Electrocatalysts Utilizing Rotating Disk Electrode Technique. *J. Electrochem. Soc.* **2015**, *162* (10), F1144–F1158. https://doi.org/10.1149/2.1071509jes.
